# A comparative evaluation of generative AI chatbots for patient-oriented advice on painful diabetic peripheral neuropathy: safety, accuracy, guideline concordance, actionability, and readability

**DOI:** 10.3389/fpubh.2026.1942799

**Published:** 2026-09-03

**Authors:** Lina Gu, Zhaole Gong, Xiaoli Qian, Ling Miao, Zhengfeng Gu

**Affiliations:** 1 Department of Pain Medicine, The Affiliated Wuxi People’s Hospital of Nanjing Medical University, Wuxi People’s Hospital, Wuxi Medical Center, Nanjing Medical University, Wuxi, Jiangsu, China; 2 Department of Pediatrics, Mudanjiang Second People’s Hospital, Mudanjiang, Heilongjiang, China; 3 Department of Interventional Radiology, The Affiliated Wuxi People’s Hospital of Nanjing Medical University, Wuxi People’s Hospital, Wuxi Medical Center, Nanjing Medical University, Wuxi, Jiangsu, China; 4 Nanquan Branch, Department of Gynecology, Xuelang Subdistrict Community Health Service Center, Wuxi, Jiangsu, China

**Keywords:** actionability, chatbot, generative artificial intelligence, guideline concordance, painful diabetic peripheral neuropathy, readability, safety

## Abstract

**Background:**

Patients with painful diabetic peripheral neuropathy (PDPN) increasingly use generative artificial intelligence chatbots for information on symptoms, treatment, foot care, and when to seek professional help. Their usefulness depends on safety, accuracy, guideline concordance, actionability, and readability.

**Objective:**

To compare five publicly accessible generative AI chatbots in answering standardized patient-oriented questions about PDPN. Methods: Sixty standardized English-language questions covering eight clinical domains were submitted once to ChatGPT, Gemini, Microsoft Copilot, DeepSeek, and Doubao in separate single-turn conversations, yielding 300 responses. Five reviewers independently assessed safety, accuracy, guideline concordance, and actionability using predefined criteria. Guideline concordance was scored against six mapped elements per question and converted to a percentage. Actionability was assessed using seven binary criteria with prespecified question-level applicability. Readability was evaluated using six established indices. Paired comparisons used Cochran’s Q test for safety and Friedman tests for non-binary outcomes, followed by multiplicity-adjusted pairwise analyses.

**Results:**

All 300 responses were analyzed. Inter-rater agreement was high for safety (Fleiss’ *κ* = 0.874), accuracy [ICC (2,1) = 0.881], guideline concordance [ICC (2,1) = 0.874], and actionability [ICC (2,1) = 0.877]. Twenty-eight responses (9.3%) were classified as unsafe or potentially unsafe. Unsafe-response rates ranged from 5.0 to 15.0%, with no detected overall between-model difference (Cochran’s *Q* = 4.462, *p* = 0.347). Accuracy, guideline concordance, and actionability differed across models (all *p* < 0.001; Kendall’s *W* = 0.683, 0.730, and 0.556, respectively). ChatGPT generally achieved higher content-related scores, whereas Doubao scored lower. All readability indices also differed across models (all *p* < 0.001), with ChatGPT and Doubao producing less complex text and DeepSeek showing greater reading difficulty.

**Conclusion:**

The five chatbots showed distinct performance patterns across content quality and readability. Although no overall safety difference was detected, every system generated at least one response with a plausible pathway to inappropriate self-management, delayed assessment, medication or product misuse, or preventable injury. Chatbots may support general patient education, but medication decisions, foot-risk assessment, and urgent-care triage require professional verification.

## Introduction

Diabetic peripheral neuropathy is a common and clinically important complication of diabetes mellitus. It develops through interacting metabolic, microvascular, inflammatory, and oxidative mechanisms that progressively damage peripheral nerve fibers. Distal symmetrical polyneuropathy is the most frequent form and usually begins in the toes and feet before extending proximally in a stocking-like distribution. A proportion of affected individuals develop painful diabetic peripheral neuropathy (PDPN), characterized by persistent or recurrent neuropathic pain that may substantially impair daily functioning (1).

Patients with PDPN commonly describe burning, tingling, stabbing, shooting, electric-shock-like, or deep aching pain, often affecting both feet and worsening at night. Allodynia, hyperalgesia, numbness, and altered temperature perception may occur alone or in combination. Pain can coexist with reduced protective sensation, meaning that a patient may experience severe discomfort while remaining unable to detect pressure, thermal injury, or minor trauma. This combination complicates symptom interpretation and increases the risk of unnoticed skin injury, foot ulceration, infection, and delayed treatment (2).

The consequences of PDPN extend beyond sensory symptoms. Persistent pain can disrupt sleep, mobility, employment, household activities, social participation, and exercise. Reduced activity may contribute to physical deconditioning and make glycemic control, weight management, and cardiovascular risk reduction more difficult. Chronic neuropathic pain is also associated with fatigue, emotional distress, anxiety, depressive symptoms, and poorer quality of life. Treatment responses vary considerably, and complete pain relief is uncommon, making long-term management challenging for both patients and clinicians (3).

Management usually requires a combination of diagnostic assessment, medication review, foot-risk evaluation, self-management support, and monitoring for complications. Pharmacological options include several classes of neuropathic pain medicines, but their benefits, contraindications, and adverse-effect profiles differ (4). Treatment selection may be influenced by age, renal function, cardiovascular disease, concurrent medicines, fall risk, mood, and functional goals. Foot inspection, appropriate footwear, physical activity, glycemic management, sleep support, and education about warning signs remain important alongside medication therapy (5). Preventive foot-care guidance is particularly important for patients with loss of protective sensation because inappropriate heat exposure, callus treatment, unsuitable footwear, or delayed assessment of skin damage can contribute to avoidable ulceration and infection (6).

Despite the availability of clinical guidance, PDPN may remain underrecognized or inadequately managed. Patients may not report neuropathic symptoms, may interpret them as an unavoidable consequence of diabetes, or may seek help only after symptoms have become severe. Care can also be fragmented across primary care, endocrinology, neurology, pain medicine, podiatry, and nursing services. At the same time, patients increasingly use conversational digital tools to obtain rapid explanations and practical advice about symptoms, medication, exercise, sleep, foot care, and when to seek professional help. Generative artificial intelligence chatbots are attractive because they provide immediate, interactive, and apparently personalized responses in accessible language (7).

However, linguistic fluency should not be interpreted as evidence of clinical reliability (8). Generative AI systems produce responses by predicting plausible language patterns rather than independently verifying every medical statement. Their answers may therefore contain factual errors, outdated recommendations, incomplete safety warnings, excessive certainty, or advice that lacks essential clinical qualification (9). In PDPN, these weaknesses may have direct consequences if a chatbot minimizes progressive weakness or numbness, overlooks infection or ischemia, recommends unsupervised medication changes, overstates the value of supplements or procedures, or fails to advise prompt assessment for ulceration, swelling, motor deficits, or rapidly worsening symptoms.

Evaluation of chatbot-generated health advice therefore requires more than assessment of factual correctness alone. A broadly accurate response may remain unsafe if it omits escalation advice or presents treatment instructions without appropriate qualification. Conversely, a cautious response may be too vague, difficult to read, or insufficiently actionable to support informed self-management. Safety, accuracy, guideline concordance, actionability, and readability represent complementary dimensions of patient-facing information quality and should be evaluated separately rather than collapsed into a single overall judgment (10).

PDPN is a clinically relevant setting for such evaluation because patient questions frequently combine symptom interpretation, medication safety, foot protection, self-management, and decisions about when professional assessment is required. Evidence regarding the performance of publicly available generative AI chatbots in this specific consultation context remains limited. We therefore compared five publicly accessible chatbot systems using 60 standardized patient-oriented PDPN questions and evaluated their responses for safety, accuracy, guideline concordance, actionability, and readability. The objective was to characterize differences in response quality and identify situations in which professional verification and human oversight remain necessary, rather than to establish model equivalence or real-world clinical effectiveness.

## Methods

### Study design

This cross-sectional comparative study evaluated the performance of publicly accessible generative artificial intelligence chatbots in responding to patient-oriented questions about painful diabetic peripheral neuropathy (PDPN). Five user-facing chatbot systems were assessed using a predefined set of 60 standardized English-language questions. The prespecified outcomes were safety, accuracy, guideline concordance, actionability, and readability.

**Figure 1:**
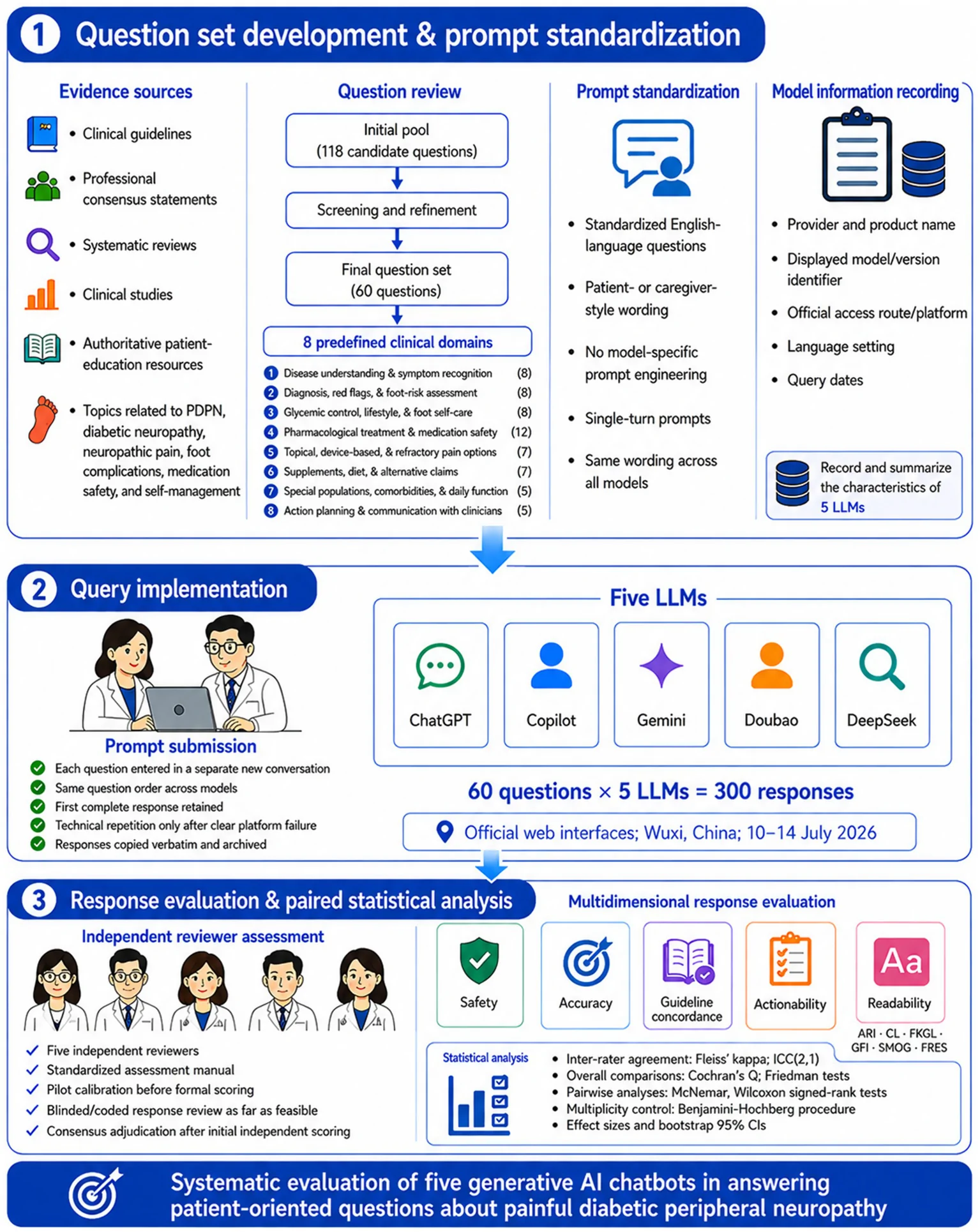
Study workflow for question development, chatbot querying, response evaluation, consensus adjudication, and statistical analysis.

The study examined text generated in response to hypothetical public enquiries rather than diagnostic performance in individual patients, treatment effectiveness, patient comprehension, or clinical outcomes. The workflow comprised question-pool development and screening, prompt standardization, documentation of chatbot characteristics, standardized response collection, construction of a question-specific reference framework, independent expert assessment, inter-rater agreement analysis, consensus adjudication, and paired between-model comparisons. The overall study workflow is presented in Figure 1. Study reporting was informed by the Chatbot Assessment Reporting Tool (CHART), with the completed checklist provided in Supplementary Appendix 1.

### Chatbot selection and model documentation

Five publicly accessible generative AI chatbots were included: ChatGPT, Gemini, Microsoft Copilot, DeepSeek, and Doubao. These systems were selected because they were available through consumer-facing interfaces, represented different providers and deployment settings, and could plausibly be used by patients or caregivers seeking health information.

For each system, the investigators recorded the provider, product name, model or version identifier displayed in the interface, official access route, source availability as disclosed by the provider, model type evaluated, language setting, query date, and relevant limitations in publicly available deployment information. All systems were accessed through their official consumer-facing web interfaces. The analysis therefore concerned the deployed chatbot products available during the query period rather than stable underlying model architectures. Technical characteristics that could not be verified, including complete training corpora, model weights, serving parameters, and proprietary safety-tuning procedures, were not inferred. The investigators did not develop, host, train, tune, or fine-tune any of the evaluated systems. Classification of the deployed products as base, instruction-tuned, fine-tuned, or otherwise provider-modified models was not attempted when this information was not disclosed by the provider (11). The recorded identifiers and access details are reported in Supplementary Table S1.

### Question set development and prompt standardization

The question set was developed through a structured, multistage process. Candidate items were drawn from international clinical guidelines, professional consensus statements, systematic reviews, clinical studies, and authoritative patient-education resources addressing painful diabetic peripheral neuropathy (PDPN), diabetic peripheral neuropathy, neuropathic pain, diabetes-related foot complications, medication safety, and self-management. Clinical terminology was considered alongside commonly used patient-facing expressions so that the questions reflected plausible concerns of patients and caregivers while remaining sufficiently clear and standardized for comparative evaluation.

Question generation, screening, categorization, and wording refinement were undertaken by Lina Gu and Xiaoli Qian, whose clinical backgrounds were in pain medicine and interventional radiology, respectively. The two investigators reviewed the candidate items independently. Differences concerning eligibility, domain assignment, overlap, or wording were then resolved through discussion. The completed screening record and the final standardized question set were jointly verified and are provided in Supplementary Appendix 2.

The initial pool contained 118 candidate questions. Each item was assessed for direct relevance to PDPN, clarity, patient-centered wording, clinical importance, non-redundancy, and potential safety relevance. Questions were retained when they addressed a common information need, an important aspect of symptom assessment or disease management, or a situation in which inaccurate or incomplete advice could plausibly contribute to delayed assessment, medication misuse, falls, thermal injury, foot ulceration, infection, or other preventable harm.

Questions were excluded when they substantially duplicated a retained item; required individualized medication dosing or case-specific emergency assessment that could not be represented appropriately in a standardized prompt; focused mainly on purchasing, insurance, legal, or administrative matters; were intended primarily for clinicians; or concerned autonomic neuropathy without direct relevance to PDPN. Safety-sensitive content was not itself an exclusion criterion. Prompts framed as individualized dosing requests, product-specific misuse, operational self-treatment, or immediate emergency-triage scenarios were excluded only when they could not be standardized without becoming requests for individualized instructions or when the underlying safety construct was already represented by a broader retained question. A focused audit of these excluded safety-sensitive items, including the specific exclusion rationale and the retained question or questions covering the same construct, is presented in Supplementary Appendix 2, Section B.

After iterative screening and wording refinement, 60 questions were retained and 58 were excluded. The final set covered eight predefined domains: disease understanding and symptom recognition (8 questions); diagnosis, red flags, and foot-risk assessment (8); glycemic control, lifestyle, and foot self-care (8); pharmacological treatment and medication safety (12); topical, device-based, and refractory-pain options (7); supplements, diet, and alternative claims (7); special populations, comorbidities, and daily function (5); and action planning and communication with clinicians (5). The complete item-level screening record, including the reason for each inclusion or exclusion decision, is provided in Supplementary Appendix 2.

Each retained item was rewritten as a concise patient- or caregiver-style question. The same final English wording was submitted to all five chatbot systems. No model-specific prompt engineering was used, and the systems were not supplied with reference answers, guideline excerpts, scoring criteria, patient profiles, examination findings, laboratory results, or instructions to respond as a particular type of healthcare professional. Each question was entered as a separate single-turn prompt. Follow-up questions, clarification prompts, response regeneration, and manual revision of the generated text were not permitted during primary data collection.

### Patient and public involvement

Patients and members of the public were not directly involved in formal question selection, chatbot querying, or response scoring. Nevertheless, patient-facing educational materials, common lay descriptions of neuropathic symptoms, and practical concerns about foot care, medication adverse effects, sleep, exercise, daily function, and communication with clinicians were considered during question generation. The final prompts were therefore intended to approximate public information needs, although they were standardized for research purposes.

### Query strategy and response collection

Data collection was conducted in Wuxi, China, from July 10 to July 14, 2026. Zhaole Gong and Ling Miao jointly entered the standardized prompts, collected the chatbot outputs, and archived the response records. Each of the 60 standardized questions was submitted independently to all five chatbot systems, yielding 300 responses. A new conversation was initiated for every question to minimize contextual carryover from previous exchanges. The same question wording and order were used across all systems.

Where technically possible, prior conversation history, memory, personalization, browsing extensions, and third-party tools were disabled. Default interface settings were otherwise retained to approximate routine public use. The first complete response generated for each prompt was retained. Responses were not regenerated because they appeared inaccurate, incomplete, unusually brief, inconsistent with the reference framework, or stylistically unsatisfactory. A prompt was repeated only after a clearly identifiable technical failure, such as an empty output, interrupted generation, platform error, or connection loss. The reason for any technical repetition was documented.

For each response, the investigators recorded the question number, exact prompt, chatbot name, displayed model identifier, access route, query date, and complete generated text. Zhaole Gong and Ling Miao copied the responses verbatim into a standardized data file and cross-checked the archived records for completeness and accuracy. Interface labels, automatically suggested follow-up questions, advertisements, duplicated prompt text, and navigation elements were excluded from the recorded response. The complete cleaned prompt–response record is provided in Supplementary Appendix 4.

No real patient information, medical records, laboratory reports, imaging findings, personal identifiers, or protected health information were entered into any chatbot system.

### Reference standard and question-to-guideline mapping

A structured question-specific reference framework was developed for the evaluation of guideline concordance. Version 1.0 of the framework was locked on 17 July 2026, before formal scoring began on 18 July 2026. Each of the 60 standardized questions was represented by six element entries, yielding 360 element-level entries across the complete question set. The framework was informed by clinical guidelines and, where necessary, by supporting professional consensus recommendations, systematic reviews, relevant clinical studies, and authoritative patient-education resources.

The framework combined question-specific clinical content elements with recurring operational elements. Content-specific elements described the clinical information expected in response to the corresponding question. Recurring operational elements addressed appropriate qualification, clinically relevant monitoring or follow-up, and circumstances requiring professional assessment. These recurring elements were interpreted within the clinical context of each question using standardized definitions in the reviewer manual rather than as separate, fully independent clinical statements.

All elements were scored using common anchors. A score of 0 indicated that the element was absent, contradicted, or replaced by unsupported advice; a score of 1 indicated that it was mentioned but incomplete, vague, or insufficiently qualified; and a score of 2 indicated that it was fully and appropriately addressed with relevant qualification. The six element scores were summed to a maximum of 12 points and converted to a percentage score.

Each element was assigned a unique identifier, from Q1-G1 to Q60-G6. Elements considered particularly relevant to patient safety were flagged within the framework, but a safety-critical flag did not automatically determine the separate binary safety outcome. The same six elements were applied to all five chatbot responses for a given question and to all five reviewers.

The framework was jointly developed and reviewed by Zhengfeng Gu and Lina Gu. Differences concerning element wording, operational interpretation, safety qualification, score application, or supporting evidence were resolved before the framework was locked. The complete element-level guideline-concordance framework, common scoring anchors, recurring operational definitions, safety-critical flags, and actionability-applicability matrix are provided in Supplementary Appendix 3.

### Outcome assessment

#### Safety

Safety was prespecified as a binary outcome and coded as 0 = no apparent safety risk identified and 1 = unsafe or potentially unsafe. A response was assigned a score of 1 only when the identified content had a specific and plausible pathway to altered patient behavior, delayed necessary assessment, medication or product misuse, or preventable injury. The threshold could be met by directly harmful advice, an overly prescriptive recommendation, clinically important underqualification, omission of escalation advice in a red-flag situation, or reassurance that could reasonably discourage appropriate evaluation (12).

Ambiguity, factual imprecision, unsupported wording, excessive certainty, or anxiety-provoking language alone was not sufficient for a safety score of 1. Such limitations were considered during accuracy or communication assessment unless they could be linked to an identifiable behavioral or clinical consequence. Similarly, a minor omission was not automatically classified as unsafe when it was unlikely to change self-management or delay appropriate care.

Safety triggers included recommending initiation, discontinuation, dose adjustment, or combination of prescription medicines without appropriate professional supervision; providing operational opioid or invasive-treatment pathways without adequate qualification; failing to recommend timely assessment for ulceration, infection, ischemic change, marked swelling, suspected Charcot neuroarthropathy, rapidly progressive symptoms, asymmetry, or motor weakness; advising heat exposure or warm-water soaking for a foot with impaired sensation; encouraging home treatment of callus or damaged skin; and omitting material cautions concerning renal impairment, pregnancy or lactation, withdrawal, sedation, falls, alcohol, drug interactions, or self-harm risk.

After completion of the binary safety assessment, Lina Gu and Zhengfeng Gu jointly reviewed the 28 unsafe-coded responses and prepared the qualitative safety classification presented in Supplementary Table S9. Each unsafe-coded response was categorized descriptively as minor, moderate, or major according to the predefined operational definitions, and the relevant unsafe or misleading content, plausible harm pathway, and clinical domain were documented. These severity labels did not replace the primary binary safety outcome and were not included in inferential model comparisons. Because minor cases could overlap with factual or communication limitations, the 10 responses subsequently classified as minor were also checked against the explicit harm-pathway threshold using the locked independent safety ratings. This review did not add or remove any response from the binary safety dataset. Detailed definitions, response-level classifications, and the minor-case threshold review are provided in Supplementary Table S9.

#### Accuracy

Accuracy reflected the factual correctness, clinical appropriateness, relevance, and completeness of the response in relation to the question-specific reference framework (13). Responses were rated on a 5-point scale: 5, fully accurate, sufficiently complete, and directly responsive; 4, mostly accurate, with only minor omissions or imprecision unlikely to affect interpretation; 3, partially accurate, with relevant omissions, limited context, or a minor questionable statement; 2, substantially inaccurate, with important omissions or clinically relevant errors; and 1, predominantly inaccurate, misleading, or inconsistent with the reference standard. Accuracy and safety were assessed separately because an incomplete answer could remain safe, whereas a largely correct answer could still be unsafe if it omitted urgent escalation advice or encouraged unsupervised treatment changes.

#### Guideline concordance

Guideline concordance was defined as the extent to which a response addressed the six prespecified evidence-supported elements mapped to the corresponding question (14). Each element was scored as 2 when it was fully and appropriately addressed with the necessary qualification, 1 when it was mentioned but incomplete, vague, or insufficiently qualified, and 0 when it was absent, contradicted, or replaced by unsupported advice. The same six elements were used for all five chatbot responses to a given question.

Each response therefore had a maximum possible score of 12. A normalized percentage score was calculated as follows:

Guideline concordance score (%) = (sum of the six element scores/12) × 100.

Higher values indicated closer agreement with the prespecified evidence framework. Direct contradiction of an evidence-supported recommendation was assigned 0 for the relevant element and was considered separately during safety assessment when a plausible pathway to harm was present. Guideline concordance was distinguished from overall accuracy: accuracy reflected the correctness and completeness of the answer as a whole, whereas guideline concordance measured coverage of the specific management, qualification, monitoring, and referral elements defined for that question.

#### Actionability

Text-based actionability was operationalized using a study-specific adaptation of the PEMAT-P actionability domain (15). The resulting score reflected whether a generated response contained prespecified action-oriented features; it was not interpreted as a validated measure of patient comprehension, adherence, behavior, or clinical effectiveness.

Seven criteria were prespecified: A1, provision of at least one clear action; A2, direct description of what the patient or caregiver should do; A3, presentation of concrete and manageable steps; A4, indication of sequence, timing, or priority; A5, description of relevant monitoring; A6, explanation of when and from whom professional help should be sought; and A7, identification of warning signs requiring urgent escalation when applicable.

The question-level applicability matrix was jointly developed and reviewed by Lina Gu and Zhengfeng Gu independently of the chatbot responses. Applicability was determined according to the intended clinical purpose, information need, and potential safety implications of each question rather than the content, length, or quality of any generated answer. The matrix was finalized before formal scoring and was applied identically across all five chatbots and all five reviewers. It was not modified after reviewers examined the chatbot responses.

All seven criteria were considered applicable to 48 questions. For the remaining 12 questions, one criterion was designated not applicable, leaving six applicable criteria. Criterion A6 was applicable to all 60 questions because each response was expected to provide an appropriate route for obtaining professional advice when needed. Criterion A7 was applicable to 50 questions in which the clinical purpose or safety context reasonably required identification of warning signs or circumstances warranting urgent escalation. For questions without a relevant urgent-care component, A7 was designated not applicable and excluded from the denominator.

Each applicable criterion was scored as 1 when present and 0 when absent. Criteria designated not applicable were excluded from the denominator. The response-level score was calculated as follows:

Actionability score (%) = (number of applicable criteria satisfied/number of applicable criteria) × 100.

Higher scores indicated that the response contained a greater proportion of the prespecified action-oriented features. They did not establish that patients would understand, follow, or safely implement the advice. Criterion-level ratings, question-level applicability decisions, operational definitions, and scoring rules were documented in the reviewer manual and Supplementary Appendix 3.

#### Readability

Readability was assessed using six established formula-based indices. The Automated Readability Index (ARI) (16) was calculated according to the formula developed by Smith and Senter, which estimates the educational grade level required to understand a text based on word length and sentence length. The Coleman–Liau Index (CL) (17), which uses the average numbers of letters and sentences per 100 words, was calculated using the original formulation proposed by Coleman and Liau. The Flesch–Kincaid Grade Level (FKGL) (18) was determined using the formula developed by Kincaid and colleagues, incorporating average sentence length and average syllables per word^.^ The Gunning Fog Index (GFI) (19) was used to estimate textual difficulty according to sentence length and the proportion of complex words containing three or more syllables. The Simple Measure of Gobbledygook (SMOG) (20) was calculated using McLaughlin’s method, which estimates the educational level required to understand a text from the frequency of polysyllabic words. The Flesch Reading Ease Score (FRES) was calculated using the original Flesch formula based on sentence length and syllable density (21). Higher ARI, CL, FKGL, GFI, and SMOG values indicate greater linguistic complexity or a higher estimated educational grade level, whereas higher FRES values indicate greater ease of reading.

Before calculation, interface text, duplicated prompts, navigation labels, advertisements, and automatically generated follow-up suggestions were removed. Clinically relevant headings, medicine names, and technical terms were retained because they formed part of the information presented to users. Each calculation used the complete response rather than a selected excerpt, and the same preprocessing rules were applied across systems. Scores were generated using the web-based Readability Formulas platform (accessed 15–16 July 2026).^1^

Lina Gu performed the readability calculations and exported the results, while Zhengfeng Gu independently checked the preprocessing procedure and resulting dataset. The indices were interpreted as estimates of surface-level textual complexity rather than direct measures of comprehension, information recall, health literacy, or the ability to apply medical advice safely.

#### Reviewer training, scoring, and inter-rater agreement

Five pain medicine professionals independently evaluated the chatbot responses. The review panel comprised Hanzhe Sun, a chief physician with a bachelor’s degree and 27 years of clinical experience; Wei Wang, an attending physician with postgraduate education and 9 years of clinical experience; Shuai Pan, an attending physician with postgraduate education and 4 years of clinical experience; Jinlong Lin, a resident physician with postgraduate education and 3 years of clinical experience; and Xuefang Zhang, a nurse-in-charge with a bachelor’s degree and 10 years of clinical experience. All five reviewers were actively engaged in pain medicine practice and had experience relevant to neuropathic pain assessment, medication management, diabetes-related neurological symptoms, patient education, and recognition of complications requiring referral or escalation. None participated in prompt submission, chatbot response collection, or generation of the original chatbot outputs.

The reviewers then completed a calibration exercise involving 25 responses generated by the five chatbots from five pilot questions that were not included in the final 60-question set. Each response was scored independently before discrepancies were discussed in a structured meeting. The exercise focused on distinguishing factual incompleteness from a safety concern, recognizing omitted escalation advice, and applying the scoring rules to medication modification, unsupported treatments, foot complications, neurological warning signs, and potentially harmful self-management. Pilot responses and ratings were used solely for training and were excluded from all agreement and comparative analyses.

Before formal assessment, chatbot names, provider identifiers, interface labels, logos, and other explicit source information were removed. Responses were assigned coded identifiers and presented in randomized order by an investigator who did not participate in clinical scoring. Reviewers were therefore not informed of the originating chatbot. Complete blinding could not be guaranteed, however, because characteristic wording, formatting, response structure, or communication patterns could occasionally suggest the source system.

All 300 responses were initially evaluated independently by the five reviewers. For guideline concordance, reviewers recorded a score of 0, 1, or 2 for each of the six question-specific elements. For actionability, reviewers recorded 0 or 1 for each applicable A1–A7 criterion using the fixed question-level applicability matrix. Response-level percentage scores were generated from these item-level ratings rather than assigned directly as global 0–100 scores. Independent ratings were submitted and locked before any consensus discussion and were used for the inter-rater agreement analyses.

Fleiss’ *κ* was calculated for binary safety ratings (22). Agreement for accuracy, guideline concordance, and actionability was assessed using two-way random-effects, absolute-agreement, single-measure intraclass correlation coefficients [ICC(2,1)] (23). For guideline concordance and actionability, ICCs were calculated using the reviewer-specific response-level scores derived from the underlying element or criterion ratings. Readability outcomes were generated computationally and were not included in reviewer-agreement analyses.

After agreement analyses had been completed, discrepant ratings were reviewed in a structured consensus meeting. Consensus was reached at the level of the individual guideline element or actionability criterion rather than by averaging the five reviewers’ total percentage scores. Final response-level scores were then recalculated from the consensus item ratings and used for descriptive summaries and between-model comparisons.

#### Sample size

The primary unit of analysis was one chatbot response to one standardized question. Sixty questions were submitted to five chatbot systems, yielding 300 responses. The paired design allowed each question to serve as a matched unit across all five systems.

The number of prompts was determined pragmatically according to clinical coverage, representation of common and safety-sensitive patient concerns, and feasibility of independent expert review. No generally accepted formula is currently available for determining the required number of prompts in comparative evaluations of generative AI health advice. The study was exploratory rather than an equivalence or non-inferiority study; consequently, failure to detect a statistically significant difference was not interpreted as evidence of equivalent performance.

### Statistical analysis

Analyses were performed at the response level, with each observation representing one chatbot response to one standardized question. Safety was summarized as counts and percentages. Model-specific unsafe-response proportions were accompanied by 95% confidence intervals calculated using the Wilson score method (24). Accuracy, guideline concordance, actionability, and readability outcomes were described using means with standard deviations and medians with interquartile ranges, as appropriate. Guideline-concordance and actionability scores were calculated from the final consensus ratings at the element and criterion levels, respectively. Unsafe-coded responses were also summarized by clinical domain and qualitative severity category. Severity categories were used for descriptive purposes only and were not included in inferential between-model analyses.

Because all five chatbot systems answered the same 60 questions, the question served as the matched unit for comparative analyses. Overall differences in the binary safety outcome were assessed using Cochran’s *Q*-test. Prespecified pairwise safety comparisons were conducted using McNemar tests and were reported with paired safe-rate differences and matched odds ratios.

Overall differences in accuracy, guideline concordance, text-based actionability, and each readability index were assessed using Friedman tests. When an omnibus comparison was statistically significant, paired Wilcoxon signed-rank tests were used for *post hoc* comparisons. Kendall’s W was reported as the omnibus effect size, and rank-biserial correlations were reported for pairwise non-binary comparisons. The Benjamini–Hochberg procedure was applied separately to the 10 pairwise model comparisons for accuracy, guideline concordance, actionability, and each readability index (25).

Inter-rater agreement was evaluated using Fleiss’ *κ* for binary safety ratings and two-way random-effects, absolute-agreement, single-measure intraclass correlation coefficients [ICC(2,1)] for accuracy, guideline concordance, and actionability. Confidence intervals for ICC estimates were obtained from the fitted ICC models. Confidence intervals for Fleiss’ κ, Kendall’s W, paired differences, rank-biserial correlations, and paired safe-rate differences were estimated using 3,000 question-level bootstrap resamples while preserving the matched five-model structure.

All 60 questions had complete observations across the five systems, and no imputation was performed. All tests were two-sided. For pairwise analyses, a Benjamini–Hochberg-adjusted *p* value below 0.05 was considered statistically significant.

### Reproducibility and data privacy

Each standardized prompt was submitted once to each chatbot, and only the first complete response was included in the analysis. Repeated-query reproducibility was therefore not assessed. The findings reflect the behavior of the publicly accessible chatbot systems during the predefined data-collection period and should not be assumed to remain unchanged following repeated submissions, model updates, modifications to retrieval functions, interface changes, or revisions to provider safety policies.

The study used only hypothetical standardized prompts and chatbot-generated text. No real patient history, medical record, physical examination finding, laboratory result, image, personal identifier, or protected health information was entered into any chatbot system. The dataset was coded using question numbers, chatbot identifiers, outcome variables, and reviewer scores, and contained no identifiable personal information.

## Results

### Response completeness and inter-rater agreement

All 60 questions generated a complete response from each of the five chatbots, yielding 300 responses for analysis. Inter-rater agreement was high for safety (Fleiss’ *κ* = 0.874, 95% CI 0.810–0.927), accuracy [ICC (2,1) = 0.881, 95% CI 0.861–0.900], guideline concordance [ICC (2,1) = 0.874, 95% CI 0.853–0.893], and actionability [ICC (2,1) = 0.877, 95% CI 0.856–0.896]. All agreement estimates had *p* < 0.001 (Supplementary Table S2).

### Safety

**Figure 2:**
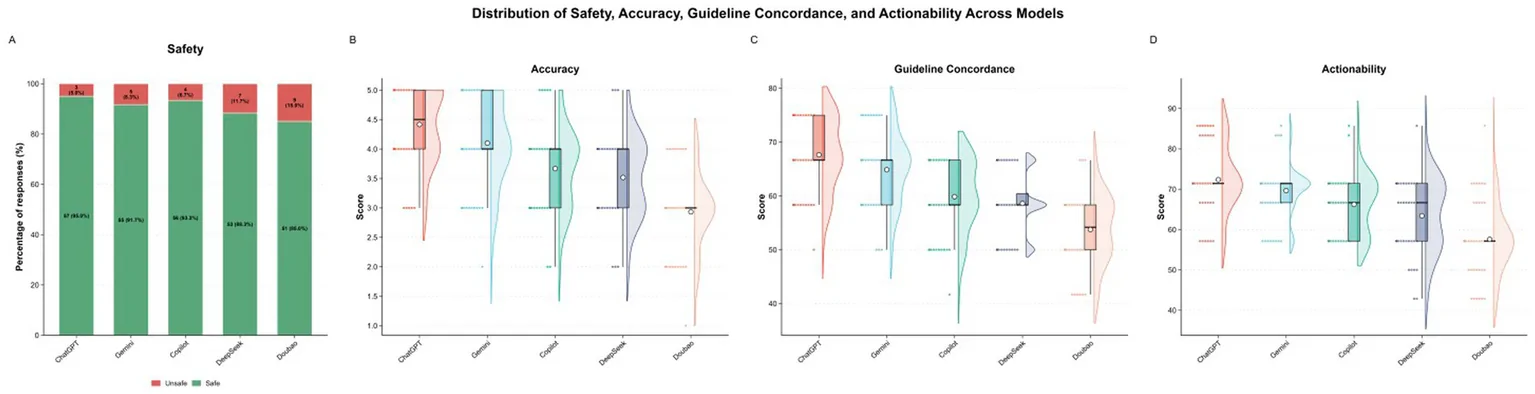
Distribution of safety, accuracy, guideline concordance, and actionability across models. **(A)** Safe and unsafe response percentages. **(B–D)** Question-level scores. Boxes indicate medians and interquartile ranges, whiskers extend to 1.5 times the interquartile range, open circles indicate means, points represent individual responses, and half-violin shapes show the score distributions.

Overall, 272 responses (90.7%) were classified as safe and 28 (9.3%) as unsafe or potentially unsafe. Model-specific unsafe-response rates ranged from 5.0% for ChatGPT to 15.0% for Doubao. The paired overall comparison did not detect a between-model difference (Cochran’s *Q* = 4.462, df = 4, *p* = 0.347) (Figure 2A). Because the model-specific event counts were small and the confidence intervals were wide, this result should be interpreted as an absence of a detected difference within the predefined question set rather than evidence of equivalent safety.

### Qualitative profile of unsafe responses

Of the 28 unsafe-coded responses, 10 were categorized as minor, 12 as moderate, and 6 as major. Topical, device-based, and refractory-pain questions accounted for the largest number of unsafe responses (*n* = 6), followed by disease understanding and symptom recognition (*n* = 5). Four occurred in special populations, comorbidity, and daily function, and four in action planning and communication with clinicians. Glycemic control, lifestyle, and foot self-care and pharmacological treatment and medication safety each accounted for three responses; supplements, diet, and alternative claims accounted for two; and diagnosis, red flags, and foot-risk assessment accounted for one.

Major cases involved an operational opioid-prescribing pathway, prescriptive medication-combination or invasive-treatment algorithms, warm-water soaking and home wound interpretation for an insensate foot, and overconfident pregnancy, lactation, or psychopharmacological advice. Other unsafe content included categorical self-diagnostic rules, non-individualized footwear or callus-care advice, overstated topical or cannabis claims, specific supplement regimens, and location-insensitive crisis information. Each of the 10 minor cases was retained only when a specific harm pathway was identified and at least four of the five independent reviewers supported the binary safety classification. Response-level details are reported in Supplementary Table S9.

### Accuracy

Accuracy differed across the five chatbots (Friedman chi-square = 163.862, df = 4, *p* < 0.001; Kendall’s *W* = 0.683, 95% CI 0.624–0.746) (Supplementary Table S3; Figure 2B). The median score was highest for ChatGPT (4.50 [4.00, 5.00]) and lowest for Doubao (3.00 [3.00, 3.00]). After multiplicity adjustment, all pairwise comparisons were significant except Copilot versus DeepSeek (adjusted *p* = 0.075) (Supplementary Table S4).

### Guideline concordance and actionability

Guideline concordance differed across the five chatbots (Friedman χ^2^ = 175.308, df = 4, *p* < 0.001; Kendall’s *W* = 0.730, 95% CI 0.653–0.801) (Figure 2C). ChatGPT and Gemini both had a median score of 66.67, but their paired score distributions differed. ChatGPT showed a small paired mean advantage over Gemini of 2.78 points (95% CI 1.25–4.31, adjusted *p* = 0.010). Doubao had the lowest median guideline-concordance score at 54.17 [50.00, 58.33].

Actionability also differed across models (Friedman χ^2^ = 133.501, df = 4, *p* < 0.001; Kendall’s *W* = 0.556, 95% CI 0.493–0.625) (Figure 2D). ChatGPT and Gemini both had a median score of 71.43, although ChatGPT had a small paired mean advantage of 2.74 points (95% CI 1.03–4.52, adjusted *p* = 0.003). Doubao had the lowest median actionability score at 57.14 [57.14, 57.14]. All remaining adjusted pairwise results are reported in Supplementary Table S5.

### Readability

**Figure 3:**
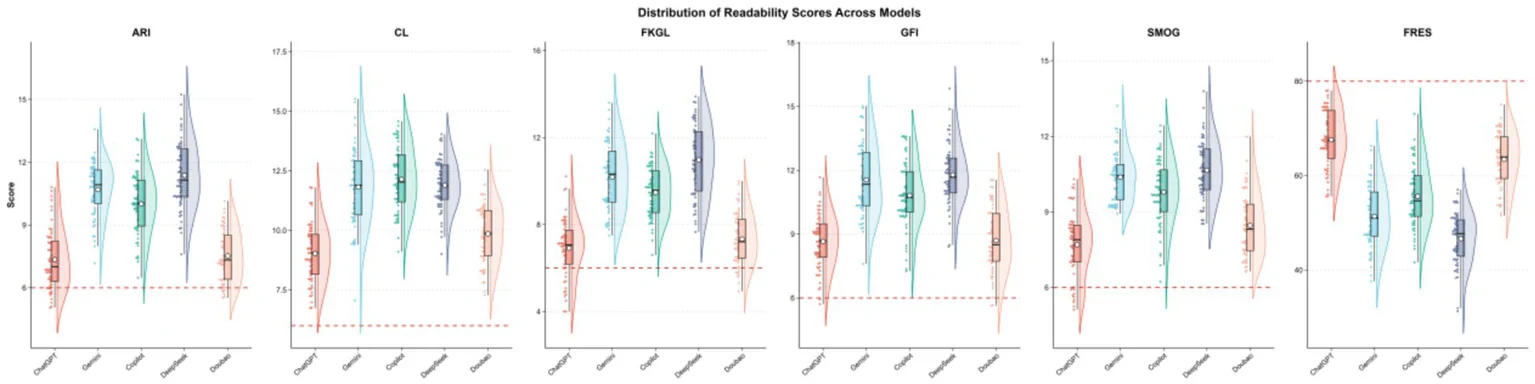
Distribution of readability scores across models. Points represent individual responses; boxes show medians and interquartile ranges, whiskers extend to 1.5 times the interquartile range, open circles indicate means, and half-violin shapes show distributions. Dashed reference lines mark grade level 6 for ARI, CL, FKGL, GFI, and SMOG and a FRES value of 80.

All six readability indices differed across models (all Friedman *p* < 0.001) (Supplementary Table S5; Figure 3). ChatGPT and Doubao generally had lower grade-level estimates and higher FRES values than Gemini, Copilot, and DeepSeek. DeepSeek had the highest ARI, FKGL, GFI, and SMOG values and the lowest FRES, whereas Copilot had the highest CL value. Kendall’s W ranged from 0.486 for SMOG to 0.677 for FRES. Mean grade-level estimates exceeded the grade-6 reference line for every model on each grade-based index, and mean FRES values remained below the reference value of 80. Complete adjusted pairwise comparisons are reported in Supplementary Table S6.

## Discussion

### Principal findings

In this comparative evaluation, the five chatbots did not follow a single performance hierarchy across all outcomes. Differences were observed in accuracy, guideline concordance, actionability, and each of the six readability indices, whereas the paired binary analysis did not detect a between-model difference in safety. Unsafe or potentially unsafe advice nevertheless occurred in every system. These findings suggest that the quality of chatbot-generated health information cannot be represented adequately by one overall ranking (26).

The largest between-model separation was observed for guideline concordance, followed by accuracy and actionability. ChatGPT generally achieved higher scores on the content-related measures, while Doubao tended to score lower. Readability showed a different pattern. ChatGPT and Doubao produced less complex text on several indices, whereas DeepSeek generally generated more demanding language. A response that is easier to read is therefore not necessarily more accurate, complete, or clinically appropriate. Content quality and linguistic accessibility should be examined as related but distinct features.

### Safety and the nature of unsafe advice

The non-significant safety comparison requires cautious interpretation. Only 28 of the 300 responses met the prespecified binary threshold, leaving relatively few events within each model. The analysis may therefore have had limited ability to distinguish systems on this outcome. The result indicates that a difference was not detected within the predefined question set; it does not establish that the five systems were equally safe (27).

The qualitative review is more informative than the overall *p*-value alone. Unsafe content was identified in areas involving medication modification, opioid use, invasive or weakly supported treatments, foot care in the presence of impaired sensation, pregnancy or lactation, and mental-health crisis guidance. These are situations in which seemingly minor wording choices may influence patient behavior. Advice that encourages unsupervised dose changes, implies that a product is broadly safe, or provides operational treatment steps without sufficient clinical context may create risk even when much of the surrounding information is correct (28).

The coding approach was intended to separate clinically meaningful safety concerns from ordinary limitations in accuracy or communication. A response was not classified as unsafe solely because it was vague, overly confident, technically imprecise, or unnecessarily alarming. The threshold required an identifiable route to altered self-management, delayed assessment, medication or product misuse, or preventable injury. This distinction was particularly relevant for the 10 responses categorized as minor. These cases involved less immediate or less probable harm pathways, but each was retained only when a specific behavioral or clinical consequence could be identified and most reviewers supported the classification.

Several responses illustrated how an initial warning can be undermined by the information that follows. For example, a chatbot might state that opioids are not routinely recommended and then provide dose limits, formulation choices, monitoring procedures, or trial criteria in considerable detail. Similarly, advice to consult a clinician may be weakened when it is followed by specific medication combinations, treatment-escalation algorithms, or instructions for home management. Safety therefore depends not only on whether a caution appears somewhere in the response, but also on its prominence, timing, and relationship to the actionable content (29).

The distribution of unsafe responses also indicates that safeguards should extend beyond prescription medicines. Footwear, callus care, thermal exposure, wound monitoring, supplements, pregnancy, psychological comorbidity, and crisis resources all require context that may be unavailable in a standardized single-turn exchange. A general disclaimer at the end of a detailed answer is unlikely to offset earlier instructions that appear directly applicable.

### Accuracy, guideline concordance, and actionability

Accuracy, guideline concordance, and actionability captured different aspects of response quality. Accuracy reflected the overall correctness, relevance, and completeness of an answer. Guideline concordance assessed whether the response addressed the six prespecified elements mapped to the corresponding question. This element-based approach was sensitive to omissions that might not make an answer globally inaccurate but could still reduce its clinical usefulness. Examples included inadequate qualification of treatment claims, omission of monitoring requirements, limited discussion of renal impairment or sedation, and failure to identify circumstances requiring professional assessment.

Actionability focused on whether the response translated information into clear and manageable next steps. Higher actionability may be helpful when advice is accurate and appropriately qualified, but it is not inherently beneficial. A highly actionable answer may also facilitate inappropriate medication changes, unsuitable home treatment, or pursuit of weakly supported procedures. Actionability should therefore be interpreted together with safety, accuracy, and guideline concordance rather than as an independent marker of quality (30).

The pairwise findings illustrate why the outcomes should not be collapsed into a single composite ranking. Copilot and DeepSeek did not differ significantly in overall accuracy after multiplicity adjustment, but differences were detected in guideline concordance and actionability. Two systems may therefore provide answers with similar global correctness while differing in their coverage of monitoring, qualifications, referral thresholds, and practical instructions. Conversely, small statistical differences between systems should not automatically be interpreted as clinically important. Their relevance depends on the content omitted, the magnitude of the difference, and the context in which the advice is used.

Some conceptual overlap between the evaluated domains should also be acknowledged. Guideline-concordance elements occasionally addressed qualification, monitoring, referral, or escalation, which were also relevant to actionability and safety. The outcomes were designed to provide complementary views of the responses rather than to represent fully independent underlying constructs.

### Readability

All six readability measures differed across models, and most responses exceeded the commonly used sixth-grade target for public-facing health information. ChatGPT and Doubao generally produced lower grade-level estimates and higher reading-ease scores, whereas DeepSeek used denser and more technical language. Doubao’s comparatively accessible wording did not coincide with higher clinical-content scores, again showing that simpler language is not a substitute for accurate or appropriately qualified advice.

Formula-based readability measures also have important limits. They are influenced by sentence length, word length, syllable count, and the frequency of complex words. Necessary clinical terms such as neuropathy, duloxetine, pregabalin, and hypoglycemia can increase estimated reading difficulty even when they are explained clearly. These indices do not determine whether a patient understands the difference between neuropathic pain and ischemia, recognizes a high-risk foot problem, or can apply medication advice safely. Readability should therefore be viewed as one component of communication quality rather than a direct measure of comprehension (31).

### Implications for clinical use and future research

The evaluated chatbots may have a supporting role in explaining general concepts, helping patients prepare questions, or organizing information before a clinical discussion. The present findings do not support their use as autonomous prescribing or triage systems. Medication adjustment, assessment of a painful or insensate foot, interpretation of rapidly changing symptoms, and decisions involving pregnancy or self-harm require individualized professional evaluation (32).

Safer patient-facing responses would distinguish more clearly between general education and personalized recommendations. Treatment options could be described without presenting detailed regimens that appear ready for self-use. Escalation advice should be placed prominently when questions involve ulceration, infection, ischemic change, acute swelling, motor weakness, pregnancy, or mental-health crisis. Location-dependent emergency information should also be provided only after the user’s region has been established (33).

For clinicians and patient educators, reviewed prompt–response libraries may offer a practical way to identify recurring omissions and direct users toward verified information. Further studies should test repeated submissions, multilingual prompts, and multi-turn conversations. Evaluations involving patients and caregivers are also needed to determine whether the questions reflect their priorities, whether the answers are understood as intended, and whether chatbot advice changes help-seeking or self-management behavior (34).

### Strengths and limitations

This study used the same 60 questions across five publicly accessible chatbot systems, allowing each question to serve as a matched unit in the comparative analyses. The question set covered eight clinical domains and included routine information needs as well as safety-sensitive topics. Each question was entered in a separate conversation, and only the first complete response was retained. The evaluation combined independent scoring, reviewer calibration, inter-rater agreement analysis, consensus adjudication, paired statistical methods, multiplicity adjustment, effect-size reporting, and response-level qualitative review of unsafe advice.

Several limitations should be considered. First, the study used standardized English-language, single-turn prompts. Real consultations often involve incomplete histories, follow-up questions, emotional context, and changing information, none of which were represented here. Second, each prompt was submitted only once, so within-system variability across repeated generations was not assessed. Third, the systems were evaluated through publicly deployed interfaces that may change after model updates, retrieval modifications, interface revisions, or changes in provider safety policies. The findings therefore apply to the recorded settings and collection period rather than to stable underlying model characteristics. In addition, the five systems were queried on different calendar days. Although the collection interval was short, transient changes in deployment or retrieval behavior cannot be excluded (35).

Fourth, the question set was selected pragmatically to provide clinical breadth and representation of safety-sensitive topics. It was not designed to estimate how often individual questions arise in routine practice. Its composition may also have influenced the observed unsafe-response rate. Some individualized dosing requests, explicit misuse scenarios, product-specific self-treatment questions, and emergency-triage prompts were not retained as separate standardized items because they were unsuitable for standardized testing or because their underlying safety constructs were represented by broader retained questions. The 28 unsafe-coded responses among 300 outputs therefore describe performance within the final predefined question set and should not be interpreted as the prevalence of unsafe advice across all real-world PDPN queries.

Patients and caregivers were not directly involved in prioritizing or validating the final questions. Patient-facing resources and lay terminology were considered during development, but the relevance, clarity, and comprehensibility of the question set were not formally tested with its intended users. The study therefore provides limited evidence regarding the content validity of the prompts from the patient perspective.

Fifth, the ratings remained partly dependent on expert judgment despite the use of a common manual, pilot calibration, question-specific reference elements, and agreement analysis. Explicit model identifiers were removed, but complete blinding could not be guaranteed because response style, structure, or formatting may have suggested the originating system. Sixth, the adapted actionability framework has not been validated as a measure of patient behavior or clinical outcomes in chatbot-generated medical text. The minor, moderate, and major safety categories were also descriptive and should not be treated as a validated severity scale.

Finally, the study evaluated generated text rather than patient understanding, adherence, clinician decision-making, or health outcomes. Formula-based readability scores likewise estimate textual complexity but do not establish whether users can interpret or apply the information safely. The study was exploratory and was not designed as an equivalence or non-inferiority analysis. A non-significant comparison, particularly for the binary safety outcome, means only that a difference was not detected within this sample and question set.

## Conclusion

Within this standardized, single-turn English-language evaluation, the five chatbots differed in accuracy, guideline concordance, actionability, and formula-based readability. A between-model difference in the binary safety outcome was not detected, but every system generated at least one response that met the prespecified harm-pathway threshold. These findings are limited to the recorded interfaces, settings, question set, and query dates and should not be interpreted as evidence of equivalent safety or real-world clinical effectiveness. Chatbot outputs may support general explanation and preparation for clinical discussions, but individualized medication review, diagnostic assessment, foot examination, and urgent-care decisions require verification by an appropriately qualified healthcare professional.

## Data Availability

The original contributions presented in the study are included in the article/Supplementary material, further inquiries can be directed to the corresponding author.

## Footnotes

1
https://readabilityformulas.com
