# Supplementary material for: A comparative evaluation of generative AI chatbots for patient-oriented advice on painful diabetic peripheral neuropathy: safety, accuracy, guideline concordance, actionability, and readability
Source: Front Public Health. 2026 Sep 3;14:1942799. doi: 10.3389/fpubh.2026.1942799 (PMC13584960; doi:10.3389/fpubh.2026.1942799)
Supplement: Supplementary file 1 [file Supplementary_file_1.docx]

**Supplementary Appendix S1. CHART Checklist**

Completed according to the manuscript: A Comparative Evaluation of Generative AI Chatbots for Patient-Oriented Advice on Painful Diabetic Peripheral Neuropathy: Safety, Accuracy, Guideline Concordance, Actionability, and Readability.

Checklist used: Chatbot Assessment Reporting Tool (CHART). Page numbers and locations refer to the revised manuscript PDF. Supplementary materials include Supplementary Tables S1-S6 and S9, and Supplementary Appendices S2-S4.

| **Heading** | **No** | **CHART checklist item** | **Reported page/location** |
| --- | --- | --- | --- |
| **Title and abstract** | | | |
| Title | 1a | State that the study is assessing one or more generative AI-driven chatbots for clinical evidence or health advice. | Title, p. 1; Abstract, pp. 2-3. The title and abstract state that the study evaluates five publicly accessible generative AI chatbots for patient-oriented health advice concerning painful diabetic peripheral neuropathy (PDPN). |
| Abstract/summary | 1b | Apply a structured format, if applicable. | Abstract, pp. 2-3. The structured abstract includes Background, Objective, Methods, Results, and Conclusions. |
| **Introduction** | | | |
| Background | 2a | State the scientific background, rationale, and healthcare context for evaluating the generative AI-driven chatbot(s), referencing relevant literature when applicable. | Introduction, pp. 4-6. The manuscript describes the clinical presentation and burden of PDPN, medication and foot-safety concerns, limitations of public health information, increasing use of conversational AI, and the rationale for multidimensional evaluation of chatbot-generated advice. |
|  | 2b | State the aims and research questions including the target audience, intervention, comparator(s), and outcome(s). | Abstract Objective, p. 2; Introduction, p. 6. Target audience: patients with PDPN, caregivers, and public users; intervention/comparators: five chatbot systems; outcomes: safety, accuracy, guideline concordance, actionability, and readability. |
| **Methods** | | | |
| Model identifiers | 3a | State the name and version identifier(s) of the generative AI model(s) and chatbot(s) under evaluation, as well as their date of release or last update. | Methods: Chatbot selection and model documentation, pp. 7-9; Supplementary Table S1. Chatbot names, displayed model/version identifiers, official access routes, query dates, and unavailable release or last-update information are reported; details not shown or independently verifiable were not inferred. |
|  | 3b | State whether the generative AI model(s) and chatbot(s) are open-source or closed-source/proprietary. | Methods: Chatbot selection and model documentation, pp. 7-9; Supplementary Table S1. Source availability and provider disclosures are recorded for the deployed products. Proprietary or undisclosed training, tuning, and serving details were reported as unavailable rather than inferred. |
| Model details | 4a | State whether the generative AI model was a base model or a novel base model, tuned model, or fine-tuned model. | Methods: Chatbot selection and model documentation, p. 9; Supplementary Table S1. The study evaluated deployed user-facing chatbot products rather than investigator-hosted model architectures. Classification as a base, instruction-tuned, fine-tuned, or otherwise provider-modified model was not attempted when this information was not disclosed. |
|  | 4b | If a base model is used, cite its development in sufficient detail to identify the model. | Not applicable. The investigators did not directly access, host, or evaluate an unmodified base model as the experimental system. Publicly deployed chatbot products were evaluated through their official web interfaces. |
|  | 4c | If a novel base model, tuned model, or fine-tuned model is used, describe the pre- and/or post-implementation/deployment data and parameters. | Not applicable. The investigators did not develop, train, tune, fine-tune, host, or deploy any model. Provider-side training data, tuning procedures, deployment parameters, and safety configurations were not publicly disclosed or independently verifiable. |
| Prompt engineering | 5a | Describe the evolution of study prompt development. | Methods: Question set development and prompt standardization, pp. 9-10; Figure 1, p. 7; Supplementary Appendix S2. The manuscript reports development of an initial pool of 118 questions, independent screening and wording refinement, exclusion of 58 items, and retention of 60 standardized questions across eight clinical domains. |
|  | 5ai | Describe the sources of prompts. | Methods: Question set development and prompt standardization, pp. 9-10; Supplementary Appendices S2-S3. Sources included international guidelines, professional consensus statements, systematic reviews, clinical studies, authoritative patient-education resources, and patient-facing terminology related to PDPN, neuropathic pain, medication safety, foot complications, and self-management. |
|  | 5aii | State the number and characteristics of the individual(s) involved in prompt engineering. | Methods: Question set development and prompt standardization, p. 9. Lina Gu, with a pain-medicine background, and Xiaoli Qian, with an interventional-radiology background, independently generated, screened, categorized, and refined the candidate questions; disagreements were resolved through discussion. |
|  | 5aiii | Provide details of any patient and public involvement during prompt engineering. | Methods: Patient and public involvement, p. 10. No patients or public representatives participated directly in formal question selection. Patient-facing educational materials, lay symptom descriptions, and practical public information needs were considered during question generation. |
|  | 5b | Provide study prompts. | Methods: Question set development and prompt standardization, pp. 9-10; Supplementary Appendix S2. The final 60 standardized English-language questions and the complete item-level screening record are provided. |
| Query strategy | 6a | State route of access to generative AI model. | Methods: Chatbot selection and model documentation and Query strategy and response collection, pp. 7-11; Supplementary Table S1. ChatGPT, Gemini, Microsoft Copilot, DeepSeek, and Doubao were accessed through their official consumer-facing web interfaces, with default settings retained except where memory, personalization, browsing extensions, or third-party tools could be disabled. |
|  | 6b | State the date(s) and location(s) of queries for the generative AI-driven chatbot(s) including the day, month, and year as well as city and country. | Methods: Query strategy and response collection, p. 10; Supplementary Table S1. Queries were conducted in Wuxi, China, from 10 to 14 July 2026. |
|  | 6c | Describe whether prompts were input into separate chat session(s). | Methods: Query strategy and response collection, pp. 10-11. A new conversation was initiated for every question to minimize contextual carryover; the same question wording and order were used across all systems. |
|  | 6d | Provide all generative AI-driven chatbot outputs/responses. | Methods: Query strategy and response collection, pp. 10-11; Supplementary Appendix S4. The first complete response was retained, copied verbatim, cross-checked, and archived. The complete cleaned prompt-response record is provided. |
| Performance evaluation | 7a | Define the ground truth or reference standard used to define successful generative AI-driven chatbot performance. | Methods: Reference standard and question-to-guideline mapping, pp. 11-12; Outcome assessment, pp. 12-15; Supplementary Appendix S3. Version 1.0 of the question-specific framework was locked on 17 July 2026 and contained 360 element-level entries, common 0-2 scoring anchors, recurring operational definitions, safety-critical flags, and a fixed actionability-applicability matrix. |
|  | 7b | Describe the process undertaken for generative AI-driven chatbot performance evaluation. | Methods: Outcome assessment and Reviewer training, scoring, and inter-rater agreement, pp. 12-16; Supplementary Appendix S3; Supplementary Table S9. The manuscript describes binary safety coding, 5-point accuracy scoring, six-element guideline-concordance scoring, seven-criterion actionability scoring, six formula-based readability indices, reviewer calibration, de-identification and randomized presentation, independent ratings, agreement analysis, and element- or criterion-level consensus adjudication. |
|  | 7bi | State the number and characteristics of team members involved in performance evaluation. | Methods: Reviewer training, scoring, and inter-rater agreement, pp. 15-16. Five pain-medicine professionals independently evaluated the responses: four physicians and one nurse-in-charge. Their names, professional ranks, educational backgrounds, and 3-27 years of clinical experience are reported. |
|  | 7bii | Provide details of any patients and public involvement during the evaluation process. | Methods: Patient and public involvement, p. 10. No patients or members of the public participated in formal chatbot querying or response scoring. |
|  | 7biii | State whether evaluators were blinded to the identity of the generative AI-driven chatbot(s) under assessment. | Methods: Reviewer training, scoring, and inter-rater agreement, p. 16. Model names, provider identifiers, interface labels, and logos were removed; responses were coded and randomized, and reviewers were not informed of the source chatbot. Complete blinding could not be guaranteed because wording, structure, or formatting might remain recognizable. |
| Sample size | 8 | Report how the sample size was determined. | Methods: Sample size, pp. 16-17. The final sample comprised 60 questions submitted to five chatbots, yielding 300 matched responses. The number of prompts was determined pragmatically by clinical coverage, representation of common and safety-sensitive concerns, and feasibility of independent expert review; no formal power calculation was used. |
| Data analysis | 9a | Describe statistical analysis methods, including any evaluation of reproducibility of generative AI-driven chatbot responses. | Methods: Statistical analysis and Reproducibility and data privacy, pp. 17-18. The manuscript reports descriptive statistics, Fleiss' kappa, ICC(2,1), Cochran's Q and McNemar tests, Friedman and paired Wilcoxon tests, Kendall's W, rank-biserial correlations, Benjamini-Hochberg correction, 3,000 matched question-level bootstrap resamples, complete-case handling, and two-sided testing. Repeated-output reproducibility was not assessed because each prompt was submitted once. |
|  | 9ai | Report the measures used for performance evaluation. | Methods: Outcome assessment and Statistical analysis, pp. 12-17. Measures were safety, accuracy, guideline concordance, actionability, Automated Readability Index, Coleman-Liau Index, Flesch-Kincaid Grade Level, Gunning Fog Index, Simple Measure of Gobbledygook, and Flesch Reading Ease Score. |
| **Results** | | | |
|  | 10a | Report the performance evaluation undertaken including the alignment between generative AI-driven chatbot output and ground truth or reference standard using quantitative or mixed methods approaches as applicable. | Results, pp. 18-22; Tables 1-5; Figures 2-3; Supplementary Tables S2-S6. Quantitative findings are reported for response completeness, inter-rater agreement, safety, accuracy, guideline concordance, actionability, and all six readability indices, with paired omnibus and multiplicity-adjusted pairwise comparisons. |
|  | 10b | For responses deviating from the ground truth or reference standard, state the nature of the difference(s). | Results: Qualitative profile of unsafe responses, p. 19; Discussion, pp. 22-25; Supplementary Tables S4-S6 and S9. Reported deviations include inadequate qualification, missing monitoring or escalation advice, unsupported or overly prescriptive treatment claims, medication and interaction concerns, unsafe foot-care suggestions, weakly supported topical or device claims, and location-insensitive crisis information. |
|  | 10c | Report the evaluation for potentially harmful, biased, or misleading responses. | Results: Safety and Qualitative profile of unsafe responses, pp. 18-19; Tables 1-2; Supplementary Tables S3 and S9. Twenty-eight of 300 responses were unsafe-coded, including 10 minor, 12 moderate, and 6 major cases; every chatbot generated at least one unsafe or potentially unsafe response. Response-level harm pathways and minor-case reviewer votes are documented. |
| **Discussion** | | | |
|  | 11a | Interpret study findings in the context of relevant evidence. | Discussion, pp. 22-25. The findings are interpreted in relation to multidimensional chatbot performance, low-event safety comparisons, medication and foot-care risks, differences between global accuracy and element-level guideline concordance, actionability, readability, and the continuing need for professional oversight. |
|  | 11b | Describe the strengths and limitations of the study. | Discussion: Strengths and limitations, pp. 25-27. Strengths include a matched 60-question design, five chatbot systems, eight clinical domains, separate conversations, first-response retention, independent scoring, calibration, agreement analysis, consensus adjudication, paired statistics, multiplicity adjustment, and response-level safety review. Limitations include English-only single-turn prompts, single submissions, dynamic interfaces, different query days, pragmatic question selection, no patient/public participation, formula-based readability measures, and no real-world clinical outcomes. |
|  | 11c | Describe the potential implications for practice, education, policy, regulation, and research. | Discussion: Implications for clinical use and future research, p. 25; Conclusion, p. 27. The manuscript supports use of chatbots only as supplementary general education, emphasizes professional verification for prescribing, foot assessment, red-flag triage, pregnancy, and self-harm concerns, and recommends clearer safety-netting, location-aware escalation, reviewed prompt-response libraries, repeated-generation testing, multilingual and multi-turn studies, and patient/caregiver evaluation. |
| **Open science** | | | |
| Disclosures | 12a | Report any relevant conflicts of interest for all authors. | Competing interests, p. 28. The authors declare that they have no competing interests. |
| Funding | 12b | Report sources of funding and their role in the conduct and reporting of the study. | Funding, p. 28. The study received no specific grant from any funding agency in the public, commercial, or not-for-profit sectors. |
| Ethics | 12c | Describe the process undertaken for ethical approval. | Ethics approval and consent to participate, p. 27. The study used standardized hypothetical prompts and chatbot-generated text and involved no human participants, identifiable personal information, medical records, biological samples, or clinical interventions. The manuscript states that, under the applicable institutional policy, it did not constitute human-participant research and did not require informed consent. |
|  | 12ci | Describe the measures taken to safeguard data privacy of patient health information, as applicable. | Methods: Reproducibility and data privacy, pp. 17-18; Data privacy, p. 27. No real patient information or protected health information was entered into any chatbot. Study files were coded by question number, chatbot, outcome, and reviewer score and contained no identifiable personal information. |
|  | 12cii | State whether permission/licensing was obtained for the use of original, copyrighted data. | Permission and licensing, p. 28. The prompts were investigator-developed from public clinical and patient-education sources. No copyrighted patient records or proprietary clinical datasets were used; sharing of chatbot outputs is subject to applicable platform terms. |
| Protocol | 12d | Provide a study protocol. | Protocol and analysis code, pp. 27-28. The study was not prospectively registered. The manuscript states that the prespecified protocol and statistical code are available from the corresponding author on reasonable request. |
| Data availability | 12e | State where study data, code repository, and model parameters can be accessed. | Availability of data and materials and Protocol and analysis code, pp. 27-28; Supplementary materials. The cleaned prompt-response dataset, finalized question bank, question-to-guideline mapping framework, reviewer manual, de-identified response-level ratings, and statistical code are available from the corresponding author on reasonable request. Proprietary model weights and serving parameters were not available to the investigators. |

Footer Note: AI, artificial intelligence; CHART, Chatbot Assessment Reporting Tool; LLM, large language model; PDPN, painful diabetic peripheral neuropathy.
