# Supplementary material for: A comparative evaluation of generative AI chatbots for patient-oriented advice on painful diabetic peripheral neuropathy: safety, accuracy, guideline concordance, actionability, and readability
Source: Front Public Health. 2026 Sep 3;14:1942799. doi: 10.3389/fpubh.2026.1942799 (PMC13584960; doi:10.3389/fpubh.2026.1942799)
Supplement: Supplementary file 5 [file Supplementary_file_5.docx]

**Supplementary Tables**

**Supplementary Table S1.**  Model identifiers and access details

| Chatbot | Provider | Model/version identifier | Access route | Source availability | Model type evaluated | Language setting | Query date | Notes |
| --- | --- | --- | --- | --- | --- | --- | --- | --- |
| ChatGPT | OpenAI | ChatGPT | Official ChatGPT web interface | Proprietary / closed-source deployed interface | User-facing deployed chatbot | English | 10July 2026 | Complete training data, model weights, and deployment parameters were not publicly available |
| Gemini 2.5 Pro | Google / Google DeepMind | Gemini 2.5 Pro | Official Gemini web interface | Proprietary / closed-source deployed interface | User-facing deployed chatbot | English | 11 July 2026 | Complete training data, model weights, and deployment parameters were not publicly available |
| Microsoft Copilot | Microsoft | Copilot | Official Microsoft Copilot web interface | Proprietary / closed-source deployed interface | User-facing deployed chatbot | English | 12 July 2026 | Underlying deployment details may vary and were recorded according to the interface available at query |
| Doubao | ByteDance | Doubao | Official Doubao web interface | Proprietary / closed-source deployed interface | User-facing deployed chatbot | English | 13 July 2026 | Complete training data, model weights, and deployment parameters were not publicly available |
| DeepSeek-V3.2-Exp | DeepSeek | DeepSeek-V3.2-Exp | Official DeepSeek web interface | Deployed chatbot interface; full deployment details not publicly available | User-facing deployed chatbot | English | 14 July 2026 | Evaluation focused on the deployed web chatbot rather than locally hosted model weights |

**Supplementary Table S2. Inter-rater agreement**

| **Metric** | **Agreement index** | **Estimate** | **95% CI** | **P value** | **Interpretation** | **Complete items** | **No. of raters** |
| --- | --- | --- | --- | --- | --- | --- | --- |
| Safety | Fleiss' kappa | 0.874 | 0.810-0.927 | <0.001 | Good | 300 | 5 |
| Accuracy | ICC(2,1) | 0.881 | 0.861-0.900 | <0.001 | Good | 300 | 5 |
| Guideline Concordance | ICC(2,1) | 0.874 | 0.853-0.893 | <0.001 | Good | 300 | 5 |
| Actionability | ICC(2,1) | 0.877 | 0.856-0.896 | <0.001 | Good | 300 | 5 |

*Note: Safety was assessed using Fleiss' kappa. Accuracy, Guideline Concordance, and Actionability were assessed using ICC(2,1).*

**Supplementary Table S3. Pairwise comparisons of safety**

| **Comparison** | **N pairs** | **Safe rate difference, %** | **Safe rate difference 95% CI** | **Safe to unsafe, n** | **Unsafe to safe, n** | **Matched OR** | **Matched OR 95% CI** | **Unadjusted P** | **Adjusted P** |
| --- | --- | --- | --- | --- | --- | --- | --- | --- | --- |
| ChatGPT vs Gemini | 60 | 3.33 | -5.00-11.67 | 4 | 2 | 2.00 | 0.37-10.92 | 0.683 | 0.966 |
| ChatGPT vs Copilot | 60 | 1.67 | -6.67-10.00 | 4 | 3 | 1.33 | 0.30-5.96 | 1.000 | 1.000 |
| ChatGPT vs DeepSeek | 60 | 6.67 | -3.33-16.67 | 7 | 3 | 2.33 | 0.60-9.02 | 0.343 | 0.966 |
| ChatGPT vs Doubao | 60 | 10.00 | -1.67-21.67 | 9 | 3 | 3.00 | 0.81-11.08 | 0.149 | 0.966 |
| Gemini vs Copilot | 60 | -1.67 | -11.67-8.33 | 4 | 5 | 0.80 | 0.21-2.98 | 1.000 | 1.000 |
| Gemini vs DeepSeek | 60 | 3.33 | -8.33-13.33 | 7 | 5 | 1.40 | 0.44-4.41 | 0.773 | 0.966 |
| Gemini vs Doubao | 60 | 6.67 | -5.00-18.33 | 9 | 5 | 1.80 | 0.60-5.37 | 0.423 | 0.966 |
| Copilot vs DeepSeek | 60 | 5.00 | -5.00-16.67 | 7 | 4 | 1.75 | 0.51-5.98 | 0.546 | 0.966 |
| Copilot vs Doubao | 60 | 8.33 | -1.67-20.00 | 8 | 3 | 2.67 | 0.71-10.05 | 0.228 | 0.966 |
| DeepSeek vs Doubao | 60 | 3.33 | -8.33-15.00 | 7 | 5 | 1.40 | 0.44-4.41 | 0.773 | 0.966 |

*Note: Pairwise comparisons were performed using McNemar tests. Adjusted P values were calculated using the Benjamini-Hochberg method. A positive safe-rate difference indicates a higher safe-response rate for the first model.*

**Supplementary Table S4. Pairwise comparisons of accuracy**

| **Metric** | **Comparison** | **N pairs** | **Mean difference** | **Mean difference 95% CI** | **Median difference** | **Median difference 95% CI** | **Rank-biserial correlation** | **Rank-biserial 95% CI** | **Unadjusted P** | **Adjusted P** |
| --- | --- | --- | --- | --- | --- | --- | --- | --- | --- | --- |
| Accuracy | ChatGPT vs Gemini | 60 | 0.32 | 0.17-0.47 | 0.00 | 0.00-0.50 | 0.704 | 0.355-1.000 | <0.001 | <0.001 |
| Accuracy | ChatGPT vs Copilot | 60 | 0.75 | 0.58-0.90 | 1.00 | 1.00-1.00 | 0.958 | 0.857-1.000 | <0.001 | <0.001 |
| Accuracy | ChatGPT vs DeepSeek | 60 | 0.90 | 0.77-1.05 | 1.00 | 1.00-1.00 | 0.965 | 0.883-1.000 | <0.001 | <0.001 |
| Accuracy | ChatGPT vs Doubao | 60 | 1.48 | 1.35-1.62 | 1.50 | 1.00-2.00 | 1.000 | 1.000-1.000 | <0.001 | <0.001 |
| Accuracy | Gemini vs Copilot | 60 | 0.43 | 0.25-0.62 | 0.00 | 0.00-1.00 | 0.804 | 0.492-1.000 | <0.001 | <0.001 |
| Accuracy | Gemini vs DeepSeek | 60 | 0.58 | 0.43-0.75 | 1.00 | 0.00-1.00 | 0.946 | 0.818-1.000 | <0.001 | <0.001 |
| Accuracy | Gemini vs Doubao | 60 | 1.17 | 1.03-1.30 | 1.00 | 1.00-1.00 | 1.000 | 1.000-1.000 | <0.001 | <0.001 |
| Accuracy | Copilot vs DeepSeek | 60 | 0.15 | 0.00-0.32 | 0.00 | 0.00-0.00 | 0.391 | 0.000-0.750 | 0.075 | 0.075 |
| Accuracy | Copilot vs Doubao | 60 | 0.73 | 0.58-0.88 | 1.00 | 1.00-1.00 | 0.957 | 0.857-1.000 | <0.001 | <0.001 |
| Accuracy | DeepSeek vs Doubao | 60 | 0.58 | 0.45-0.70 | 1.00 | 0.00-1.00 | 1.000 | 1.000-1.000 | <0.001 | <0.001 |

*Note: Paired Wilcoxon signed-rank tests were used. Positive mean differences and rank-biserial correlations indicate higher scores for the first model.*

**Supplementary Table S5. Pairwise comparisons of guideline concordance and actionability**

| **Metric** | **Comparison** | **N pairs** | **Mean difference** | **Mean difference 95% CI** | **Median difference** | **Median difference 95% CI** | **Rank-biserial correlation** | **Rank-biserial 95% CI** | **Unadjusted P** | **Adjusted P** |
| --- | --- | --- | --- | --- | --- | --- | --- | --- | --- | --- |
| Guideline Concordance | ChatGPT vs Gemini | 60 | 2.78 | 1.25-4.31 | 0.00 | 0.00-8.33 | 0.547 | 0.195-0.890 | 0.009 | 0.010 |
| Guideline Concordance | ChatGPT vs Copilot | 60 | 7.78 | 6.53-8.89 | 8.33 | 8.33-8.33 | 0.967 | 0.879-1.000 | <0.001 | <0.001 |
| Guideline Concordance | ChatGPT vs DeepSeek | 60 | 9.03 | 7.78-10.28 | 8.33 | 8.33-8.33 | 0.976 | 0.914-1.000 | <0.001 | <0.001 |
| Guideline Concordance | ChatGPT vs Doubao | 60 | 13.89 | 12.50-15.28 | 16.67 | 16.67-16.67 | 0.992 | 0.968-1.000 | <0.001 | <0.001 |
| Guideline Concordance | Gemini vs Copilot | 60 | 5.00 | 3.75-6.25 | 8.33 | 0.00-8.33 | 1.000 | 1.000-1.000 | <0.001 | <0.001 |
| Guideline Concordance | Gemini vs DeepSeek | 60 | 6.25 | 5.00-7.64 | 8.33 | 8.33-8.33 | 1.000 | 1.000-1.000 | <0.001 | <0.001 |
| Guideline Concordance | Gemini vs Doubao | 60 | 11.11 | 9.72-12.64 | 8.33 | 8.33-8.33 | 1.000 | 1.000-1.000 | <0.001 | <0.001 |
| Guideline Concordance | Copilot vs DeepSeek | 60 | 1.25 | 0.14-2.36 | 0.00 | 0.00-0.00 | 0.680 | 0.250-0.963 | 0.012 | 0.012 |
| Guideline Concordance | Copilot vs Doubao | 60 | 6.11 | 4.72-7.50 | 8.33 | 8.33-8.33 | 0.951 | 0.861-1.000 | <0.001 | <0.001 |
| Guideline Concordance | DeepSeek vs Doubao | 60 | 4.86 | 3.75-5.97 | 8.33 | 0.00-8.33 | 1.000 | 1.000-1.000 | <0.001 | <0.001 |
| Actionability | ChatGPT vs Gemini | 60 | 2.74 | 1.03-4.52 | 0.00 | 0.00-0.00 | 0.883 | 0.618-1.000 | 0.002 | 0.003 |
| Actionability | ChatGPT vs Copilot | 60 | 6.15 | 4.09-8.22 | 0.00 | 0.00-14.29 | 0.841 | 0.564-1.000 | <0.001 | <0.001 |
| Actionability | ChatGPT vs DeepSeek | 60 | 8.97 | 6.79-10.99 | 14.29 | 14.29-14.29 | 0.934 | 0.820-1.000 | <0.001 | <0.001 |
| Actionability | ChatGPT vs Doubao | 60 | 14.80 | 13.29-16.35 | 14.29 | 14.29-14.29 | 1.000 | 1.000-1.000 | <0.001 | <0.001 |
| Actionability | Gemini vs Copilot | 60 | 3.41 | 1.71-5.16 | 0.00 | 0.00-0.00 | 0.787 | 0.338-1.000 | 0.004 | 0.004 |
| Actionability | Gemini vs DeepSeek | 60 | 6.23 | 4.37-8.10 | 0.00 | 0.00-14.29 | 1.000 | 1.000-1.000 | <0.001 | <0.001 |
| Actionability | Gemini vs Doubao | 60 | 12.06 | 10.20-13.77 | 14.29 | 14.29-14.29 | 1.000 | 1.000-1.000 | <0.001 | <0.001 |
| Actionability | Copilot vs DeepSeek | 60 | 2.82 | 1.07-4.60 | 0.00 | 0.00-0.00 | 0.783 | 0.412-1.000 | 0.007 | 0.007 |
| Actionability | Copilot vs Doubao | 60 | 8.65 | 6.67-10.79 | 14.29 | 0.00-14.29 | 1.000 | 1.000-1.000 | <0.001 | <0.001 |
| Actionability | DeepSeek vs Doubao | 60 | 5.83 | 3.85-7.98 | 0.00 | 0.00-0.00 | 1.000 | 1.000-1.000 | <0.001 | <0.001 |

*Note: Paired Wilcoxon signed-rank tests were used with Benjamini-Hochberg correction. Bootstrap 95% confidence intervals are reported for paired differences and effect sizes.*

**Supplementary Table S6. Pairwise comparisons of readability outcomes**

| **Metric** | **Comparison** | **N pairs** | **Mean difference** | **Mean difference 95% CI** | **Median difference** | **Median difference 95% CI** | **Rank-biserial correlation** | **Rank-biserial 95% CI** | **Unadjusted P** | **Adjusted P** |
| --- | --- | --- | --- | --- | --- | --- | --- | --- | --- | --- |
| ARI | ChatGPT vs Gemini | 60 | -3.35 | -3.78--2.92 | -3.56 | -3.87--2.84 | -0.993 | -1.000--0.973 | <0.001 | <0.001 |
| ARI | ChatGPT vs Copilot | 60 | -2.66 | -3.16--2.17 | -2.96 | -3.36--2.04 | -0.944 | -0.992--0.866 | <0.001 | <0.001 |
| ARI | ChatGPT vs DeepSeek | 60 | -4.03 | -4.50--3.55 | -4.08 | -4.49--3.38 | -1.000 | -1.000--1.000 | <0.001 | <0.001 |
| ARI | ChatGPT vs Doubao | 60 | -0.18 | -0.62-0.28 | -0.31 | -0.64-0.18 | -0.149 | -0.439-0.142 | 0.318 | 0.318 |
| ARI | Gemini vs Copilot | 60 | 0.68 | 0.24-1.11 | 1.04 | 0.20-1.49 | 0.435 | 0.162-0.674 | 0.003 | 0.004 |
| ARI | Gemini vs DeepSeek | 60 | -0.68 | -1.15--0.21 | -0.52 | -1.12-0.11 | -0.362 | -0.615--0.081 | 0.015 | 0.017 |
| ARI | Gemini vs Doubao | 60 | 3.17 | 2.71-3.62 | 3.32 | 2.59-3.95 | 0.975 | 0.912-1.000 | <0.001 | <0.001 |
| ARI | Copilot vs DeepSeek | 60 | -1.36 | -1.85--0.86 | -1.25 | -2.18--0.84 | -0.668 | -0.867--0.422 | <0.001 | <0.001 |
| ARI | Copilot vs Doubao | 60 | 2.48 | 2.01-2.95 | 2.67 | 1.62-3.20 | 0.927 | 0.830-0.990 | <0.001 | <0.001 |
| ARI | DeepSeek vs Doubao | 60 | 3.85 | 3.32-4.37 | 4.10 | 3.10-4.48 | 0.995 | 0.976-1.000 | <0.001 | <0.001 |
| CL | ChatGPT vs Gemini | 60 | -2.79 | -3.22--2.31 | -3.02 | -3.55--2.36 | -0.932 | -0.998--0.813 | <0.001 | <0.001 |
| CL | ChatGPT vs Copilot | 60 | -3.12 | -3.61--2.61 | -3.32 | -3.71--2.48 | -0.969 | -0.997--0.914 | <0.001 | <0.001 |
| CL | ChatGPT vs DeepSeek | 60 | -2.87 | -3.28--2.46 | -2.93 | -3.40--2.35 | -0.984 | -1.000--0.945 | <0.001 | <0.001 |
| CL | ChatGPT vs Doubao | 60 | -0.83 | -1.29--0.38 | -0.83 | -1.34--0.19 | -0.465 | -0.702--0.203 | 0.002 | 0.003 |
| CL | Gemini vs Copilot | 60 | -0.33 | -0.84-0.18 | -0.18 | -0.84-0.12 | -0.186 | -0.474-0.101 | 0.211 | 0.264 |
| CL | Gemini vs DeepSeek | 60 | -0.08 | -0.61-0.46 | -0.15 | -0.69-0.83 | -0.046 | -0.346-0.233 | 0.760 | 0.760 |
| CL | Gemini vs Doubao | 60 | 1.96 | 1.52-2.42 | 1.82 | 1.40-2.46 | 0.887 | 0.765-0.969 | <0.001 | <0.001 |
| CL | Copilot vs DeepSeek | 60 | 0.25 | -0.18-0.67 | 0.06 | -0.37-0.81 | 0.144 | -0.152-0.419 | 0.335 | 0.372 |
| CL | Copilot vs Doubao | 60 | 2.29 | 1.82-2.74 | 2.19 | 1.67-2.81 | 0.931 | 0.835-0.985 | <0.001 | <0.001 |
| CL | DeepSeek vs Doubao | 60 | 2.04 | 1.63-2.46 | 1.91 | 1.52-2.68 | 0.918 | 0.808-0.991 | <0.001 | <0.001 |
| FKGL | ChatGPT vs Gemini | 60 | -3.27 | -3.75--2.80 | -3.02 | -3.84--2.64 | -0.986 | -1.000--0.953 | <0.001 | <0.001 |
| FKGL | ChatGPT vs Copilot | 60 | -2.56 | -2.93--2.18 | -2.69 | -3.10--2.23 | -0.969 | -1.000--0.911 | <0.001 | <0.001 |
| FKGL | ChatGPT vs DeepSeek | 60 | -4.06 | -4.56--3.56 | -4.67 | -5.11--3.98 | -0.989 | -1.000--0.962 | <0.001 | <0.001 |
| FKGL | ChatGPT vs Doubao | 60 | -0.42 | -0.86-0.03 | -0.34 | -0.93-0.12 | -0.268 | -0.526-0.022 | 0.072 | 0.072 |
| FKGL | Gemini vs Copilot | 60 | 0.71 | 0.26-1.13 | 0.47 | 0.13-1.28 | 0.421 | 0.158-0.657 | 0.005 | 0.006 |
| FKGL | Gemini vs DeepSeek | 60 | -0.79 | -1.42--0.15 | -1.04 | -1.90--0.47 | -0.368 | -0.632--0.096 | 0.013 | 0.015 |
| FKGL | Gemini vs Doubao | 60 | 2.85 | 2.34-3.34 | 3.13 | 2.83-3.33 | 0.946 | 0.871-0.990 | <0.001 | <0.001 |
| FKGL | Copilot vs DeepSeek | 60 | -1.50 | -1.95--1.03 | -1.75 | -2.35--1.01 | -0.737 | -0.890--0.538 | <0.001 | <0.001 |
| FKGL | Copilot vs Doubao | 60 | 2.14 | 1.70-2.58 | 2.25 | 1.74-2.79 | 0.924 | 0.822-0.991 | <0.001 | <0.001 |
| FKGL | DeepSeek vs Doubao | 60 | 3.64 | 3.13-4.15 | 3.68 | 3.23-4.09 | 0.990 | 0.961-1.000 | <0.001 | <0.001 |
| GFI | ChatGPT vs Gemini | 60 | -2.90 | -3.42--2.36 | -2.56 | -3.40--1.81 | -0.972 | -1.000--0.915 | <0.001 | <0.001 |
| GFI | ChatGPT vs Copilot | 60 | -2.16 | -2.67--1.63 | -2.31 | -2.76--1.77 | -0.863 | -0.956--0.730 | <0.001 | <0.001 |
| GFI | ChatGPT vs DeepSeek | 60 | -3.13 | -3.62--2.66 | -2.98 | -3.53--2.43 | -0.990 | -1.000--0.962 | <0.001 | <0.001 |
| GFI | ChatGPT vs Doubao | 60 | -0.05 | -0.52-0.46 | -0.36 | -0.90-0.65 | -0.022 | -0.325-0.268 | 0.886 | 0.886 |
| GFI | Gemini vs Copilot | 60 | 0.73 | 0.20-1.27 | 0.37 | -0.11-1.16 | 0.344 | 0.067-0.600 | 0.021 | 0.026 |
| GFI | Gemini vs DeepSeek | 60 | -0.24 | -0.69-0.19 | -0.31 | -0.82-0.46 | -0.143 | -0.426-0.144 | 0.339 | 0.376 |
| GFI | Gemini vs Doubao | 60 | 2.85 | 2.28-3.40 | 2.89 | 2.18-3.08 | 0.968 | 0.888-1.000 | <0.001 | <0.001 |
| GFI | Copilot vs DeepSeek | 60 | -0.97 | -1.54--0.38 | -0.78 | -1.38--0.04 | -0.433 | -0.668--0.157 | 0.004 | 0.005 |
| GFI | Copilot vs Doubao | 60 | 2.12 | 1.61-2.63 | 2.08 | 1.62-2.60 | 0.853 | 0.710-0.954 | <0.001 | <0.001 |
| GFI | DeepSeek vs Doubao | 60 | 3.09 | 2.56-3.62 | 3.06 | 2.32-3.66 | 0.973 | 0.922-0.997 | <0.001 | <0.001 |
| SMOG | ChatGPT vs Gemini | 60 | -2.68 | -3.09--2.25 | -2.46 | -3.05--1.96 | -0.991 | -1.000--0.967 | <0.001 | <0.001 |
| SMOG | ChatGPT vs Copilot | 60 | -2.09 | -2.57--1.56 | -2.05 | -2.85--1.37 | -0.846 | -0.953--0.692 | <0.001 | <0.001 |
| SMOG | ChatGPT vs DeepSeek | 60 | -2.96 | -3.38--2.53 | -2.86 | -3.43--2.26 | -0.995 | -1.000--0.977 | <0.001 | <0.001 |
| SMOG | ChatGPT vs Doubao | 60 | -0.76 | -1.20--0.32 | -0.56 | -0.92--0.07 | -0.417 | -0.651--0.146 | 0.005 | 0.006 |
| SMOG | Gemini vs Copilot | 60 | 0.59 | 0.19-1.05 | 0.34 | -0.14-1.10 | 0.342 | 0.066-0.587 | 0.022 | 0.024 |
| SMOG | Gemini vs DeepSeek | 60 | -0.28 | -0.63-0.08 | 0.03 | -0.59-0.49 | -0.175 | -0.449-0.128 | 0.245 | 0.245 |
| SMOG | Gemini vs Doubao | 60 | 1.92 | 1.58-2.26 | 1.92 | 1.43-2.55 | 0.957 | 0.892-0.993 | <0.001 | <0.001 |
| SMOG | Copilot vs DeepSeek | 60 | -0.87 | -1.32--0.45 | -0.84 | -1.45--0.32 | -0.510 | -0.728--0.261 | <0.001 | <0.001 |
| SMOG | Copilot vs Doubao | 60 | 1.32 | 0.88-1.75 | 1.71 | 1.36-2.05 | 0.706 | 0.500-0.869 | <0.001 | <0.001 |
| SMOG | DeepSeek vs Doubao | 60 | 2.19 | 1.78-2.60 | 2.40 | 1.96-2.85 | 0.925 | 0.833-0.981 | <0.001 | <0.001 |
| FRES | ChatGPT vs Gemini | 60 | 16.22 | 13.90-18.43 | 16.23 | 13.10-21.37 | 0.991 | 0.966-1.000 | <0.001 | <0.001 |
| FRES | ChatGPT vs Copilot | 60 | 11.98 | 9.53-14.41 | 12.06 | 9.88-16.46 | 0.911 | 0.811-0.976 | <0.001 | <0.001 |
| FRES | ChatGPT vs DeepSeek | 60 | 20.95 | 18.80-23.13 | 20.83 | 18.15-24.12 | 1.000 | 1.000-1.000 | <0.001 | <0.001 |
| FRES | ChatGPT vs Doubao | 60 | 4.16 | 1.99-6.35 | 4.34 | 1.59-6.85 | 0.502 | 0.249-0.729 | <0.001 | <0.001 |
| FRES | Gemini vs Copilot | 60 | -4.24 | -6.52--1.91 | -4.70 | -6.37--1.99 | -0.472 | -0.705--0.217 | 0.002 | 0.002 |
| FRES | Gemini vs DeepSeek | 60 | 4.72 | 2.86-6.76 | 5.29 | 1.55-7.28 | 0.591 | 0.343-0.789 | <0.001 | <0.001 |
| FRES | Gemini vs Doubao | 60 | -12.06 | -14.01--10.14 | -10.49 | -14.63--8.88 | -0.975 | -0.999--0.920 | <0.001 | <0.001 |
| FRES | Copilot vs DeepSeek | 60 | 8.96 | 6.85-11.05 | 7.89 | 4.93-11.34 | 0.888 | 0.767-0.975 | <0.001 | <0.001 |
| FRES | Copilot vs Doubao | 60 | -7.82 | -10.21--5.47 | -9.21 | -11.25--5.31 | -0.732 | -0.896--0.533 | <0.001 | <0.001 |
| FRES | DeepSeek vs Doubao | 60 | -16.79 | -18.57--15.03 | -16.86 | -19.71--14.45 | -1.000 | -1.000--1.000 | <0.001 | <0.001 |

*Note: For FRES, higher scores indicate easier readability; for ARI, CL, FKGL, GFI, and SMOG, lower scores indicate easier readability.*

**Supplementary Table S7. Significant pairwise comparisons for non-binary outcomes**

| **Metric** | **Comparison** | **N pairs** | **Mean difference** | **Mean difference 95% CI** | **Median difference** | **Median difference 95% CI** | **Rank-biserial correlation** | **Rank-biserial 95% CI** | **Unadjusted P** | **Adjusted P** |
| --- | --- | --- | --- | --- | --- | --- | --- | --- | --- | --- |
| Accuracy | ChatGPT vs Gemini | 60 | 0.32 | 0.17-0.47 | 0.00 | 0.00-0.50 | 0.704 | 0.355-1.000 | <0.001 | <0.001 |
| Accuracy | ChatGPT vs Copilot | 60 | 0.75 | 0.58-0.90 | 1.00 | 1.00-1.00 | 0.958 | 0.857-1.000 | <0.001 | <0.001 |
| Accuracy | ChatGPT vs DeepSeek | 60 | 0.90 | 0.77-1.05 | 1.00 | 1.00-1.00 | 0.965 | 0.883-1.000 | <0.001 | <0.001 |
| Accuracy | ChatGPT vs Doubao | 60 | 1.48 | 1.35-1.62 | 1.50 | 1.00-2.00 | 1.000 | 1.000-1.000 | <0.001 | <0.001 |
| Accuracy | Gemini vs Copilot | 60 | 0.43 | 0.25-0.62 | 0.00 | 0.00-1.00 | 0.804 | 0.492-1.000 | <0.001 | <0.001 |
| Accuracy | Gemini vs DeepSeek | 60 | 0.58 | 0.43-0.75 | 1.00 | 0.00-1.00 | 0.946 | 0.818-1.000 | <0.001 | <0.001 |
| Accuracy | Gemini vs Doubao | 60 | 1.17 | 1.03-1.30 | 1.00 | 1.00-1.00 | 1.000 | 1.000-1.000 | <0.001 | <0.001 |
| Accuracy | Copilot vs Doubao | 60 | 0.73 | 0.58-0.88 | 1.00 | 1.00-1.00 | 0.957 | 0.857-1.000 | <0.001 | <0.001 |
| Accuracy | DeepSeek vs Doubao | 60 | 0.58 | 0.45-0.70 | 1.00 | 0.00-1.00 | 1.000 | 1.000-1.000 | <0.001 | <0.001 |
| Guideline Concordance | ChatGPT vs Gemini | 60 | 2.78 | 1.25-4.31 | 0.00 | 0.00-8.33 | 0.547 | 0.195-0.890 | 0.009 | 0.010 |
| Guideline Concordance | ChatGPT vs Copilot | 60 | 7.78 | 6.53-8.89 | 8.33 | 8.33-8.33 | 0.967 | 0.879-1.000 | <0.001 | <0.001 |
| Guideline Concordance | ChatGPT vs DeepSeek | 60 | 9.03 | 7.78-10.28 | 8.33 | 8.33-8.33 | 0.976 | 0.914-1.000 | <0.001 | <0.001 |
| Guideline Concordance | ChatGPT vs Doubao | 60 | 13.89 | 12.50-15.28 | 16.67 | 16.67-16.67 | 0.992 | 0.968-1.000 | <0.001 | <0.001 |
| Guideline Concordance | Gemini vs Copilot | 60 | 5.00 | 3.75-6.25 | 8.33 | 0.00-8.33 | 1.000 | 1.000-1.000 | <0.001 | <0.001 |
| Guideline Concordance | Gemini vs DeepSeek | 60 | 6.25 | 5.00-7.64 | 8.33 | 8.33-8.33 | 1.000 | 1.000-1.000 | <0.001 | <0.001 |
| Guideline Concordance | Gemini vs Doubao | 60 | 11.11 | 9.72-12.64 | 8.33 | 8.33-8.33 | 1.000 | 1.000-1.000 | <0.001 | <0.001 |
| Guideline Concordance | Copilot vs DeepSeek | 60 | 1.25 | 0.14-2.36 | 0.00 | 0.00-0.00 | 0.680 | 0.250-0.963 | 0.012 | 0.012 |
| Guideline Concordance | Copilot vs Doubao | 60 | 6.11 | 4.72-7.50 | 8.33 | 8.33-8.33 | 0.951 | 0.861-1.000 | <0.001 | <0.001 |
| Guideline Concordance | DeepSeek vs Doubao | 60 | 4.86 | 3.75-5.97 | 8.33 | 0.00-8.33 | 1.000 | 1.000-1.000 | <0.001 | <0.001 |
| Actionability | ChatGPT vs Gemini | 60 | 2.74 | 1.03-4.52 | 0.00 | 0.00-0.00 | 0.883 | 0.618-1.000 | 0.002 | 0.003 |
| Actionability | ChatGPT vs Copilot | 60 | 6.15 | 4.09-8.22 | 0.00 | 0.00-14.29 | 0.841 | 0.564-1.000 | <0.001 | <0.001 |
| Actionability | ChatGPT vs DeepSeek | 60 | 8.97 | 6.79-10.99 | 14.29 | 14.29-14.29 | 0.934 | 0.820-1.000 | <0.001 | <0.001 |
| Actionability | ChatGPT vs Doubao | 60 | 14.80 | 13.29-16.35 | 14.29 | 14.29-14.29 | 1.000 | 1.000-1.000 | <0.001 | <0.001 |
| Actionability | Gemini vs Copilot | 60 | 3.41 | 1.71-5.16 | 0.00 | 0.00-0.00 | 0.787 | 0.338-1.000 | 0.004 | 0.004 |
| Actionability | Gemini vs DeepSeek | 60 | 6.23 | 4.37-8.10 | 0.00 | 0.00-14.29 | 1.000 | 1.000-1.000 | <0.001 | <0.001 |
| Actionability | Gemini vs Doubao | 60 | 12.06 | 10.20-13.77 | 14.29 | 14.29-14.29 | 1.000 | 1.000-1.000 | <0.001 | <0.001 |
| Actionability | Copilot vs DeepSeek | 60 | 2.82 | 1.07-4.60 | 0.00 | 0.00-0.00 | 0.783 | 0.412-1.000 | 0.007 | 0.007 |
| Actionability | Copilot vs Doubao | 60 | 8.65 | 6.67-10.79 | 14.29 | 0.00-14.29 | 1.000 | 1.000-1.000 | <0.001 | <0.001 |
| Actionability | DeepSeek vs Doubao | 60 | 5.83 | 3.85-7.98 | 0.00 | 0.00-0.00 | 1.000 | 1.000-1.000 | <0.001 | <0.001 |
| ARI | ChatGPT vs Gemini | 60 | -3.35 | -3.78--2.92 | -3.56 | -3.87--2.84 | -0.993 | -1.000--0.973 | <0.001 | <0.001 |
| ARI | ChatGPT vs Copilot | 60 | -2.66 | -3.16--2.17 | -2.96 | -3.36--2.04 | -0.944 | -0.992--0.866 | <0.001 | <0.001 |
| ARI | ChatGPT vs DeepSeek | 60 | -4.03 | -4.50--3.55 | -4.08 | -4.49--3.38 | -1.000 | -1.000--1.000 | <0.001 | <0.001 |
| ARI | Gemini vs Copilot | 60 | 0.68 | 0.24-1.11 | 1.04 | 0.20-1.49 | 0.435 | 0.162-0.674 | 0.003 | 0.004 |
| ARI | Gemini vs DeepSeek | 60 | -0.68 | -1.15--0.21 | -0.52 | -1.12-0.11 | -0.362 | -0.615--0.081 | 0.015 | 0.017 |
| ARI | Gemini vs Doubao | 60 | 3.17 | 2.71-3.62 | 3.32 | 2.59-3.95 | 0.975 | 0.912-1.000 | <0.001 | <0.001 |
| ARI | Copilot vs DeepSeek | 60 | -1.36 | -1.85--0.86 | -1.25 | -2.18--0.84 | -0.668 | -0.867--0.422 | <0.001 | <0.001 |
| ARI | Copilot vs Doubao | 60 | 2.48 | 2.01-2.95 | 2.67 | 1.62-3.20 | 0.927 | 0.830-0.990 | <0.001 | <0.001 |
| ARI | DeepSeek vs Doubao | 60 | 3.85 | 3.32-4.37 | 4.10 | 3.10-4.48 | 0.995 | 0.976-1.000 | <0.001 | <0.001 |
| CL | ChatGPT vs Gemini | 60 | -2.79 | -3.22--2.31 | -3.02 | -3.55--2.36 | -0.932 | -0.998--0.813 | <0.001 | <0.001 |
| CL | ChatGPT vs Copilot | 60 | -3.12 | -3.61--2.61 | -3.32 | -3.71--2.48 | -0.969 | -0.997--0.914 | <0.001 | <0.001 |
| CL | ChatGPT vs DeepSeek | 60 | -2.87 | -3.28--2.46 | -2.93 | -3.40--2.35 | -0.984 | -1.000--0.945 | <0.001 | <0.001 |
| CL | ChatGPT vs Doubao | 60 | -0.83 | -1.29--0.38 | -0.83 | -1.34--0.19 | -0.465 | -0.702--0.203 | 0.002 | 0.003 |
| CL | Gemini vs Doubao | 60 | 1.96 | 1.52-2.42 | 1.82 | 1.40-2.46 | 0.887 | 0.765-0.969 | <0.001 | <0.001 |
| CL | Copilot vs Doubao | 60 | 2.29 | 1.82-2.74 | 2.19 | 1.67-2.81 | 0.931 | 0.835-0.985 | <0.001 | <0.001 |
| CL | DeepSeek vs Doubao | 60 | 2.04 | 1.63-2.46 | 1.91 | 1.52-2.68 | 0.918 | 0.808-0.991 | <0.001 | <0.001 |
| FKGL | ChatGPT vs Gemini | 60 | -3.27 | -3.75--2.80 | -3.02 | -3.84--2.64 | -0.986 | -1.000--0.953 | <0.001 | <0.001 |
| FKGL | ChatGPT vs Copilot | 60 | -2.56 | -2.93--2.18 | -2.69 | -3.10--2.23 | -0.969 | -1.000--0.911 | <0.001 | <0.001 |
| FKGL | ChatGPT vs DeepSeek | 60 | -4.06 | -4.56--3.56 | -4.67 | -5.11--3.98 | -0.989 | -1.000--0.962 | <0.001 | <0.001 |
| FKGL | Gemini vs Copilot | 60 | 0.71 | 0.26-1.13 | 0.47 | 0.13-1.28 | 0.421 | 0.158-0.657 | 0.005 | 0.006 |
| FKGL | Gemini vs DeepSeek | 60 | -0.79 | -1.42--0.15 | -1.04 | -1.90--0.47 | -0.368 | -0.632--0.096 | 0.013 | 0.015 |
| FKGL | Gemini vs Doubao | 60 | 2.85 | 2.34-3.34 | 3.13 | 2.83-3.33 | 0.946 | 0.871-0.990 | <0.001 | <0.001 |
| FKGL | Copilot vs DeepSeek | 60 | -1.50 | -1.95--1.03 | -1.75 | -2.35--1.01 | -0.737 | -0.890--0.538 | <0.001 | <0.001 |
| FKGL | Copilot vs Doubao | 60 | 2.14 | 1.70-2.58 | 2.25 | 1.74-2.79 | 0.924 | 0.822-0.991 | <0.001 | <0.001 |
| FKGL | DeepSeek vs Doubao | 60 | 3.64 | 3.13-4.15 | 3.68 | 3.23-4.09 | 0.990 | 0.961-1.000 | <0.001 | <0.001 |
| GFI | ChatGPT vs Gemini | 60 | -2.90 | -3.42--2.36 | -2.56 | -3.40--1.81 | -0.972 | -1.000--0.915 | <0.001 | <0.001 |
| GFI | ChatGPT vs Copilot | 60 | -2.16 | -2.67--1.63 | -2.31 | -2.76--1.77 | -0.863 | -0.956--0.730 | <0.001 | <0.001 |
| GFI | ChatGPT vs DeepSeek | 60 | -3.13 | -3.62--2.66 | -2.98 | -3.53--2.43 | -0.990 | -1.000--0.962 | <0.001 | <0.001 |
| GFI | Gemini vs Copilot | 60 | 0.73 | 0.20-1.27 | 0.37 | -0.11-1.16 | 0.344 | 0.067-0.600 | 0.021 | 0.026 |
| GFI | Gemini vs Doubao | 60 | 2.85 | 2.28-3.40 | 2.89 | 2.18-3.08 | 0.968 | 0.888-1.000 | <0.001 | <0.001 |
| GFI | Copilot vs DeepSeek | 60 | -0.97 | -1.54--0.38 | -0.78 | -1.38--0.04 | -0.433 | -0.668--0.157 | 0.004 | 0.005 |
| GFI | Copilot vs Doubao | 60 | 2.12 | 1.61-2.63 | 2.08 | 1.62-2.60 | 0.853 | 0.710-0.954 | <0.001 | <0.001 |
| GFI | DeepSeek vs Doubao | 60 | 3.09 | 2.56-3.62 | 3.06 | 2.32-3.66 | 0.973 | 0.922-0.997 | <0.001 | <0.001 |
| SMOG | ChatGPT vs Gemini | 60 | -2.68 | -3.09--2.25 | -2.46 | -3.05--1.96 | -0.991 | -1.000--0.967 | <0.001 | <0.001 |
| SMOG | ChatGPT vs Copilot | 60 | -2.09 | -2.57--1.56 | -2.05 | -2.85--1.37 | -0.846 | -0.953--0.692 | <0.001 | <0.001 |
| SMOG | ChatGPT vs DeepSeek | 60 | -2.96 | -3.38--2.53 | -2.86 | -3.43--2.26 | -0.995 | -1.000--0.977 | <0.001 | <0.001 |
| SMOG | ChatGPT vs Doubao | 60 | -0.76 | -1.20--0.32 | -0.56 | -0.92--0.07 | -0.417 | -0.651--0.146 | 0.005 | 0.006 |
| SMOG | Gemini vs Copilot | 60 | 0.59 | 0.19-1.05 | 0.34 | -0.14-1.10 | 0.342 | 0.066-0.587 | 0.022 | 0.024 |
| SMOG | Gemini vs Doubao | 60 | 1.92 | 1.58-2.26 | 1.92 | 1.43-2.55 | 0.957 | 0.892-0.993 | <0.001 | <0.001 |
| SMOG | Copilot vs DeepSeek | 60 | -0.87 | -1.32--0.45 | -0.84 | -1.45--0.32 | -0.510 | -0.728--0.261 | <0.001 | <0.001 |
| SMOG | Copilot vs Doubao | 60 | 1.32 | 0.88-1.75 | 1.71 | 1.36-2.05 | 0.706 | 0.500-0.869 | <0.001 | <0.001 |
| SMOG | DeepSeek vs Doubao | 60 | 2.19 | 1.78-2.60 | 2.40 | 1.96-2.85 | 0.925 | 0.833-0.981 | <0.001 | <0.001 |
| FRES | ChatGPT vs Gemini | 60 | 16.22 | 13.90-18.43 | 16.23 | 13.10-21.37 | 0.991 | 0.966-1.000 | <0.001 | <0.001 |
| FRES | ChatGPT vs Copilot | 60 | 11.98 | 9.53-14.41 | 12.06 | 9.88-16.46 | 0.911 | 0.811-0.976 | <0.001 | <0.001 |
| FRES | ChatGPT vs DeepSeek | 60 | 20.95 | 18.80-23.13 | 20.83 | 18.15-24.12 | 1.000 | 1.000-1.000 | <0.001 | <0.001 |
| FRES | ChatGPT vs Doubao | 60 | 4.16 | 1.99-6.35 | 4.34 | 1.59-6.85 | 0.502 | 0.249-0.729 | <0.001 | <0.001 |
| FRES | Gemini vs Copilot | 60 | -4.24 | -6.52--1.91 | -4.70 | -6.37--1.99 | -0.472 | -0.705--0.217 | 0.002 | 0.002 |
| FRES | Gemini vs DeepSeek | 60 | 4.72 | 2.86-6.76 | 5.29 | 1.55-7.28 | 0.591 | 0.343-0.789 | <0.001 | <0.001 |
| FRES | Gemini vs Doubao | 60 | -12.06 | -14.01--10.14 | -10.49 | -14.63--8.88 | -0.975 | -0.999--0.920 | <0.001 | <0.001 |
| FRES | Copilot vs DeepSeek | 60 | 8.96 | 6.85-11.05 | 7.89 | 4.93-11.34 | 0.888 | 0.767-0.975 | <0.001 | <0.001 |
| FRES | Copilot vs Doubao | 60 | -7.82 | -10.21--5.47 | -9.21 | -11.25--5.31 | -0.732 | -0.896--0.533 | <0.001 | <0.001 |
| FRES | DeepSeek vs Doubao | 60 | -16.79 | -18.57--15.03 | -16.86 | -19.71--14.45 | -1.000 | -1.000--1.000 | <0.001 | <0.001 |

*Note: Only comparisons remaining significant after Benjamini-Hochberg correction are shown.*

**Supplementary Table 8. Significant pairwise comparisons of safety**

**Note**

No significant pairwise safety difference after Benjamini-Hochberg correction.

*Note: Only comparisons remaining significant after Benjamini-Hochberg correction are shown.*
